# Increasing structural and functional complexity in self-assembled coordination cages

**DOI:** 10.1039/d1sc01226f

**Published:** 2021-05-10

**Authors:** Sonja Pullen, Jacopo Tessarolo, Guido H. Clever

**Affiliations:** Department of Chemistry and Chemical Biology, TU Dortmund University Otto-Hahn-Straße 6 44227 Dortmund Germany jacopo.tessarolo@tu-dortmund.de guido.clever@tu-dortmund.de; Homogeneous, Supramolecular and Bio-Inspired Catalysis, Van't Hoff Institute for Molecular Sciences, University of Amsterdam Science Park 904 1098 XH Amsterdam The Netherlands s.pullen@uva.nl

## Abstract

Progress in metallo-supramolecular chemistry creates potential to synthesize functional nano systems and intelligent materials of increasing complexity. In the past four decades, metal-mediated self-assembly has produced a wide range of structural motifs such as helicates, grids, links, knots, spheres and cages, with particularly the latter ones catching growing attention, owing to their nano-scale cavities. Assemblies serving as hosts allow application as selective receptors, confined reaction environments and more. Recently, the field has made big steps forward by implementing dedicated functionality, *e.g.* catalytic centres or photoswitches to allow stimuli control. Besides incorporation in homoleptic systems, composed of one type of ligand, desire arose to include more than one function within the same assembly. Inspiration comes from natural enzymes that congregate, for example, a substrate recognition site, an allosteric regulator element and a reaction centre. Combining several functionalities without creating statistical mixtures, however, requires a toolbox of sophisticated assembly strategies. This review showcases the implementation of function into self-assembled cages and devises strategies to selectively form heteroleptic structures. We discuss first examples resulting from a combination of both principles, namely multicomponent multifunctional host–guest complexes, and their potential in application in areas such as sensing, catalysis, and photo-redox systems.

## Abstract 2

This review highlights recent strategies towards the rational synthesis of metallo-supramolecular multicomponent systems, the implementation of functionality and the challenge to create multifunctional assemblies in non-statistical fashion.

## Graphical abstract

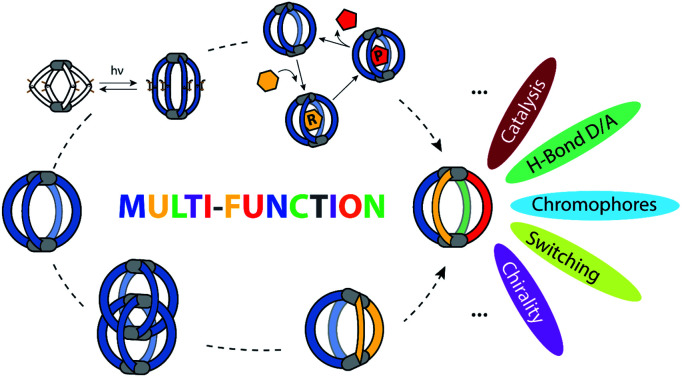

## 1. Introduction

The self-assembly of discrete nanoscale host architectures such as rings, cages and spheres has evolved into a vibrant subfield of supramolecular chemistry over the last decades.^1–4^ Routes to assemble such compounds can be primarily subclassified into hydrogen-bonded,^5,6^ dynamic-covalent^7–12^ and metal-mediated approaches.^13–28^ In the latter category, most systems are built up from a combination of two major components: metal centres acting as nodes, and organic ligands serving as bridges to join the nodes into a regularly shaped, 3-dimensional object with an accessible cavity. Metal nodes are mostly from the transition elements, but systems based on main group metals have been reported as well.^29^ Organic bridges are multitopic ligands with two or more donors of same or differing chemical structure and a backbone that determines size and shape of the assembly and can carry extra functional or solubilizing groups. While the individual donor groups can be mono- or multidentate, ligands are usually designed not to chelate the same metal so they serve as bridges between different metal nodes (a recent notable exception is a cavity formed by a pair of mechanically entangled “figure-eight”-shaped Pd^II^-chelate complexes^30^).

In order to create discrete, monodisperse products, a defined curvature is introduced within the structure of the organic bridge, the geometry of the coordination sphere, or both. Following fundamental mathematical principles, the formed shape of the hollow object is in most cases highly symmetric with all ligands adopting uniform positions around the central void space.

While this leads to beautiful structures, *e.g.* ones resembling the Platonic, Archimedean or similar solids,^31,32^ the ability to only incorporate one type of organic ligand at a time puts a limit on equipping these cages with advanced functionality such as selective recognition and chemical transformations inside the cavity. In particular when comparing self-assembled cages to their often-called natural paradigms – protein-based enzymes – one realizes that their complexity differs vastly.^33,34^ Cavities around the active sites of enzymes are of low symmetry, chiral, and contain a mix of different chemical functionalities such as recognition sites, catalytic groups and conformational switches.

**Fig. 1:**
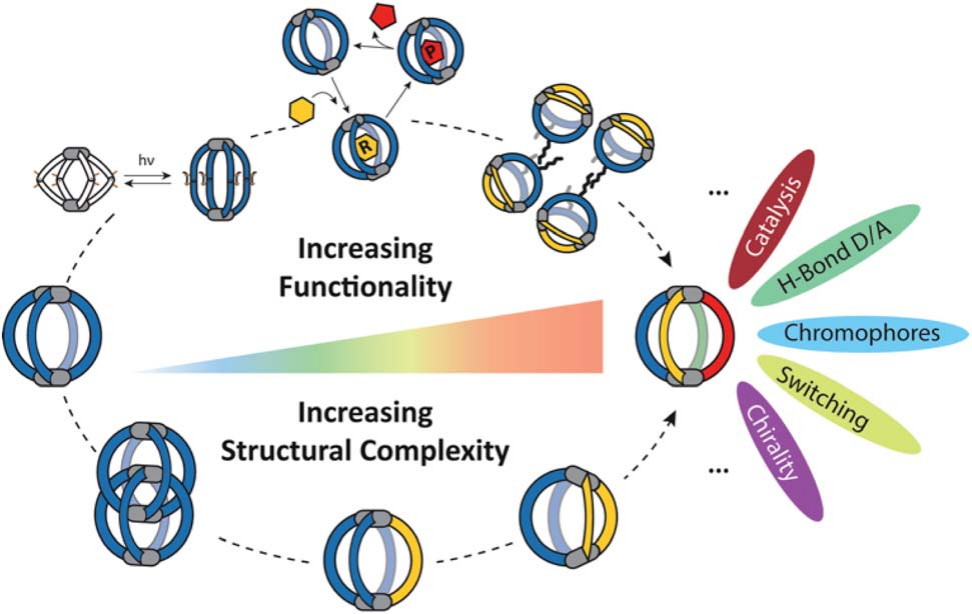
Increasing structural complexity and functionality of self-assembled coordination cages (organic ligands in colour, metal nodes as grey pills).

Can this structural and functional complexity be mimicked by artificial self-assembled structures? Differently functionalized ligands of otherwise same shape and dimensions can certainly be mixed and subjected to metal-mediated (or dynamic covalent) assembly. However, this usually leads to complex product mixtures governed by a statistical distribution of the components within the structures.^35–37^ In contrast, in this review we focus on strategies for the rational introduction of multiple building blocks into metal-mediated architectures. Our discussion will be held on two different levels (Fig. 1): on the one hand, we present a critical discussion on synthetic approaches for the integrative, non-statistical formation of a single desired assembly product from a variety of individual components. We set focus on the assembly of heteroleptic coordination cages, *i.e.* structures containing two or more unequal ligands. We distinguish between different synthetic strategies (*e.g.* shape complementarity, coordination sphere engineering, exploitation of secondary interactions…) and illustrate these principles with examples from the recent literature. On the other hand, we examine the introduction of functionality into supramolecular cages. Here, most reported examples up to now still feature only one single functionality, two functions but in statistical fashion, or one function based on a ligand's chemistry and another resulting from the entire assembly (*e.g.* guest uptake) or the metal node. So far, the non-statistical combination of structural complexity in terms of heteroleptic assembly with two or more distinguishable functionalities, acting together to create an emergent property, has rarely been reported.^38^ While we cover a few examples in this review (and most reported are rather rings than cages), the controlled implementation of multiple functionalities into self-assembled, 3-dimensional nano confinements is a research field in its infancy, showing the potential to yield a new level of supramolecular devices and materials in the future. Within this review, we highlight developments in this direction with a choice of examples from the literature, including some our own laboratory's work. As we could not cover all so far reported studies comprehensively, we apologize for what might not have found entry into this selection and refer to further reviews in this direction.^26,39–43^

## 2. Increasing structural complexity

Coordination cages are constructed from single metal nodes or, sometimes, small metal clusters that are bridged by multitopic ligands. Numerous homoleptic coordination cages have been reported in the literature and well-studied in terms of structure, host–guest dynamics and reaction enhancement.^44–48^ Most assembly strategies for such systems are based on nodes that either consist of *cis*- or *trans*-protected transition metal cations or “naked” metal ions whose coordination spheres are fully occupied by the donors of the bridging ligands.^38^ The desire to increase the complexity of such systems by combining two or more different ligands (or metals) to give one individual supramolecular object has motivated researchers to develop sophisticated strategies for heteroleptic (or heterometallic) assembly.^42,49–51^ While the synthesis of heteroleptic structures based on the *cis*- or *trans*-protected metal nodes has already advanced to high levels, with numerous examples from Stang,^52^ Fujita,^53^ Mukherjee^54^ and others, the exploration of heteroleptic assembly based on unprotected metal cations such as Pd^II^ has only recently been successful.^36,38^

Certainly, heteroleptic coordination cages can be easily obtained by simply mixing different ligands with similar size and shape. This, however, leads to statistical mixtures that may serve certain applications quite well but usually lack unambiguous spectroscopic signatures. This complicates drawing clear structure-function relationships. In order to achieve better control over stoichiometry, stereochemistry and structure of such assemblies, the development of methods for the non-statistical formation of only one desired product from a mixture of different individual building blocks is essential.

**Fig. 2:**
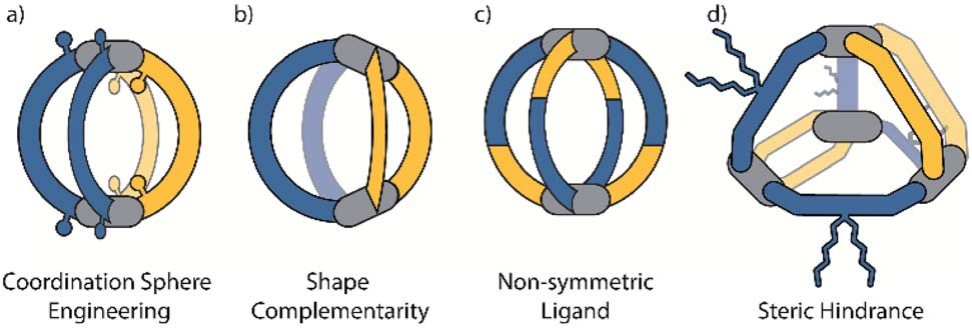
Schematic illustration of the four strategies for achieving heteroleptic cages in a non-statistical fashion: (a) coordination sphere engineering (CSE), (b) shape complementary assembly (SCA), (c) assembly from non-symmetric ligands and (d) backbone-centred steric hindrance.

In the following discussion we focus on four different approaches that have been used for the controlled assembly of non-statistical heteroleptic coordination cages: (a) coordination sphere engineering (CSE), (b) shape complementary assembly (SCA), (c) non-symmetric ligands and (d) backbone-centred steric hindrance (Fig. 2). These strategies can also be combined with each other. In addition to the combination of two or more different ligands, structural complexity can also be achieved when the same ligand is sitting in different positions within a single supramolecular cage. We include a brief discussion on such kind of structures at the beginning.

In order to favour integrative self-sorting, *i.e.* to avoid either narcissistic orthogonality or forming statistical mixtures of all possible constituents but sort the system towards one single target-compound, energetic benefits arising from enthalpic and entropic effects have to be considered.^55^ In this sense, certain structural features and supramolecular interactions can either be used to destabilize the homoleptic species, or to specifically stabilize the heteroleptic compound, thus leading to a clean and predictable assembly outcome. The most important goal in this respect is to overcome the entropic drive to produce random ligand combinations which is often a major hurdle when working with dynamically self-assembling species in thermodynamic equilibrium.

### 2.1 Homoleptic systems with ligands adopting different positions

**Fig. 3:**
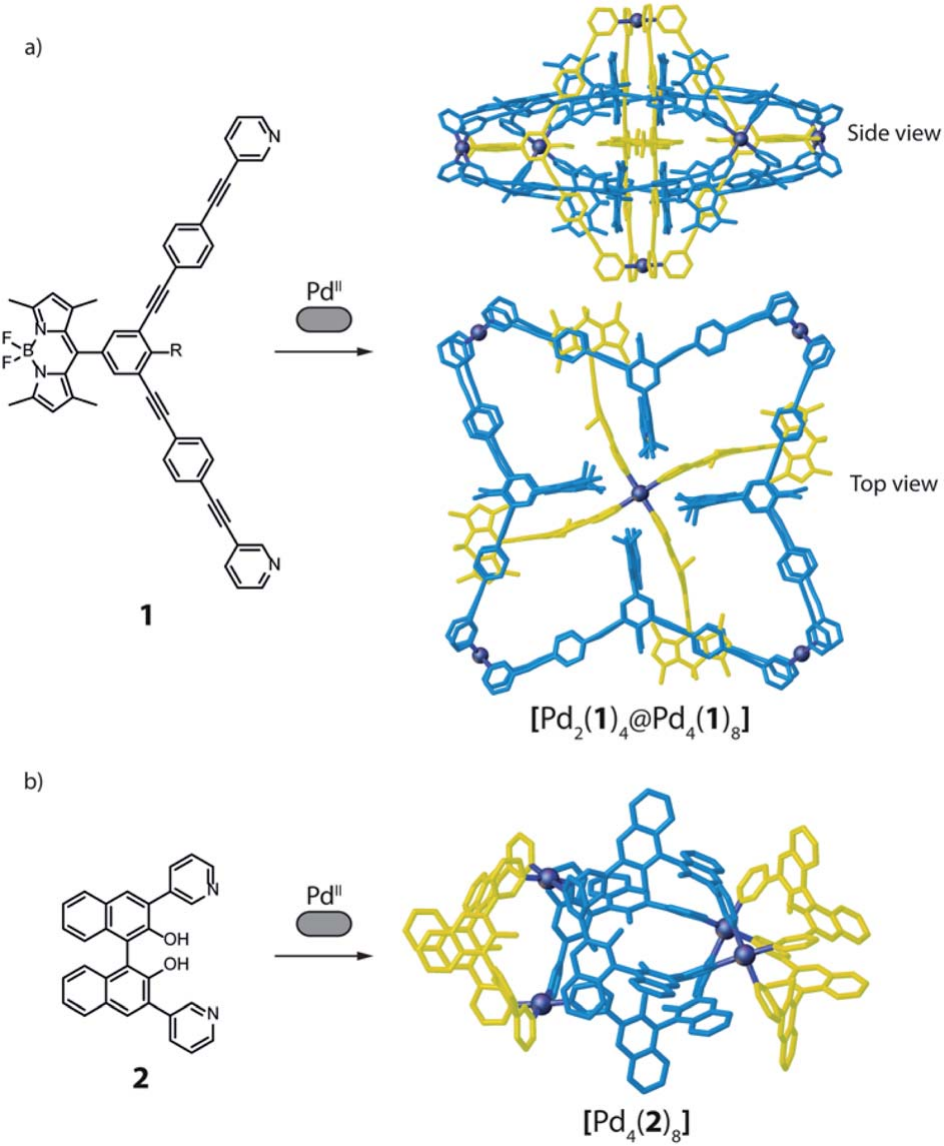
(a) Ligand **1** self-assembles with Pd^II^ cations to give a cage-in-ring architecture, where **1** occupies two different positions; (b) distorted tetrahedron obtained from a mixture of Pd^II^ and **2**, where the ligand positions are not equivalent.

A surprising degree of structural complexity can already be achieved by using only one type of ligand and metal (homoleptic species) when the same ligand occupies different positions in the resulting metallo-supramolecular structure, or when interlocked structures are formed, resulting in desymmetrisation of the ligand. Interlocking as key structural motif was described for a variety of interpenetrated cages,^56,57^ resulting in multi-cavity assemblies with different nuclearities and topologies, such as [Pd_4_L_8_] double cages,^56,57^ a [Pd_6_L_8_] peanut-shaped system,^58^ a huge [Pd_8_L_16_] catenane,^59^ or a [Pd_12_L_6_] conjoined twin-cage.^60^ Recently, we contributed a [Pd_2_**L**_4_] host with a new mechanically-interlocked motif, consisting of a pair of doubly-interlocked, figure-eight-shaped lemniscates.^30^ Even higher complexity was reported by Lützen and co-workers, with a rotaxane-like cage-in-ring structural motif, where a BODIPY-based bis-pyridine ligand (**1**) self-assembles with Pd^II^ cations to form a compound of [Pd_6_**1**_12_] stoichiometry (Fig. 3a). The structure was identified as entirely new motif that consists of both a lantern-shaped [Pd_2_**1**_4_] cage and a [Pd_4_**1**_8_] ring species, where the cage is embedded in the centre of the ring and stabilized by means of π–π interactions.^61^ A further example of homoleptic structures with the same ligand occupying different positions is found among the possible topologies resulting from the [Pd_4_L_8_] stoichiometry. Besides 4-membered rings and interpenetrated dimers,^4^ one further conceivable structure is a tetrahedron-like arrangement in which four edges consists of one single ligand, while the remaining two edges are double bridged by two ligands.^62–64^ Again, Lützen and co-workers were the ones to report an example of such a tetrahedral [Pd_4_L_8_] compound, albeit a strongly distorted, that relies on an anion templating effect. Four of its chemically identical ligands (**2**) adopt a C-shaped conformation whereas the other four adopt a twist-folded W-shaped arrangement (Fig. 3b).^65^

These examples demonstrate how relatively high structural complexity can already be achieved by using only one type of ligand. However, it is desirable to combine more than one type of ligand in selective and predictable ways as this would allow to construct multifunctional systems. In the following sections we will focus on approaches to form such heteroleptic structures using “naked” metal ions, meaning that all coordination sites on the metal nodes are occupied by donor groups of the bridging ligands.

### 2.2. Coordination sphere engineering

A first concept that has been used to prepare heteroleptic metallo-supramolecular cages is the so-called coordination sphere engineering (CSE) approach. This strategy relies on a specific design of the donor sites surrounding the metal centre. For instance, ligands with large substituents close to the donor site increase steric hindrance around the metal centre, thus influence its coordination behaviour and favour the heteroleptic combination with less bulky ligands. Alternatively, functional groups that contribute to weak interactions (*e.g.* hydrogen bonds) can be added, allowing to foster the formation of heteroleptic over homoleptic species due to a specific enthalpic gain that can only be realized by the combination of different ligands around the metal nodes. These concepts have been widely studied for *cis*-protected metal ions.^43,49^ More recent studies in the literature demonstrate how this approach can be applied to systems based on “naked” metal ions that shall be the focus of this review. In case of the popular square-planar Pd^II^ cations, a balanced choice of donor combinations can lead – in the simplest dinuclear assemblies – to the formation of heteroleptic *cis*- or *trans*-configured [M_2_L^A^_2_L^B^_2_] cages, the controlled assembly of [M_2_L_3_Solv_2_] bowl-shaped structures (Solv = coordinating solvent) or even [M_2_L_2_Solv_4_] rings.

**Fig. 4:**
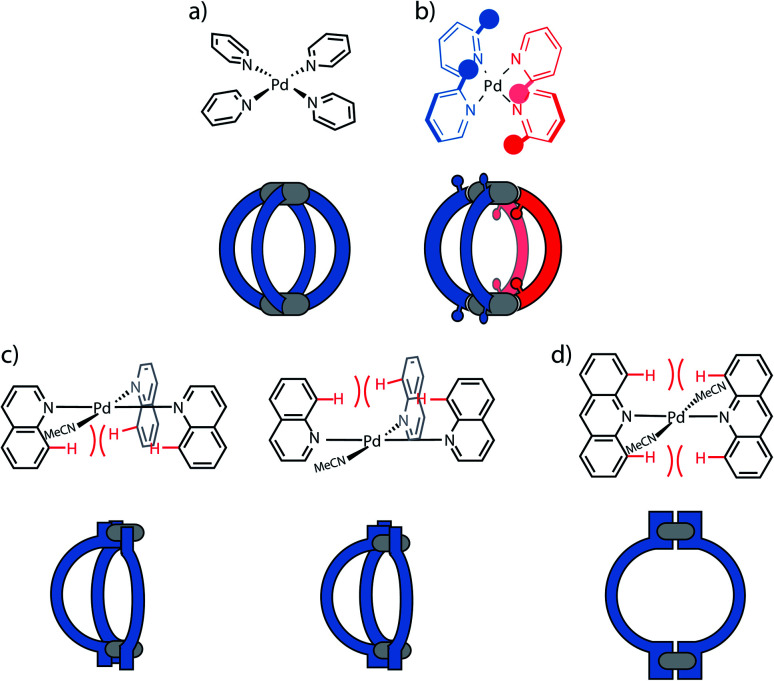
Coordination sphere engineering approaches developed for square-planar Pd^II^ systems. (a) Pd^II^ cations form with four pyridine-based ligands, devoid of steric hindrance, standard homoleptic [Pd_2_**L**_4_] cages; (b) the 1 : 1 combination of picolyl ligands with substituents in 3- and 5-positions leads to heteroleptic [Pd_2_L^A^_2_L^B^_2_] cages; (c) quinoline donors are characterized by steric repulsion between neighbouring H atoms (red colour) and allow to form bowl-like [Pd_2_L_3_Solv_2_] species (two isomeric forms shown); (d) naphthyridine donors increase steric hindrance even further and lead to the formation of [Pd_2_L_2_Solv_4_] rings.

The latter two can act as platforms for the formation of even more complex heteroleptic species (Fig. 4 and 27): While steric congestion around the metal ion prevents further bulky ligands from saturating the coordination sphere and the remaining “free” positions are occupied by coordinating solvent molecules, these may then be replaced by ligands presenting less steric bulk and higher coordinative strength. The following examples serve to illustrate these principles.

**Fig. 5:**
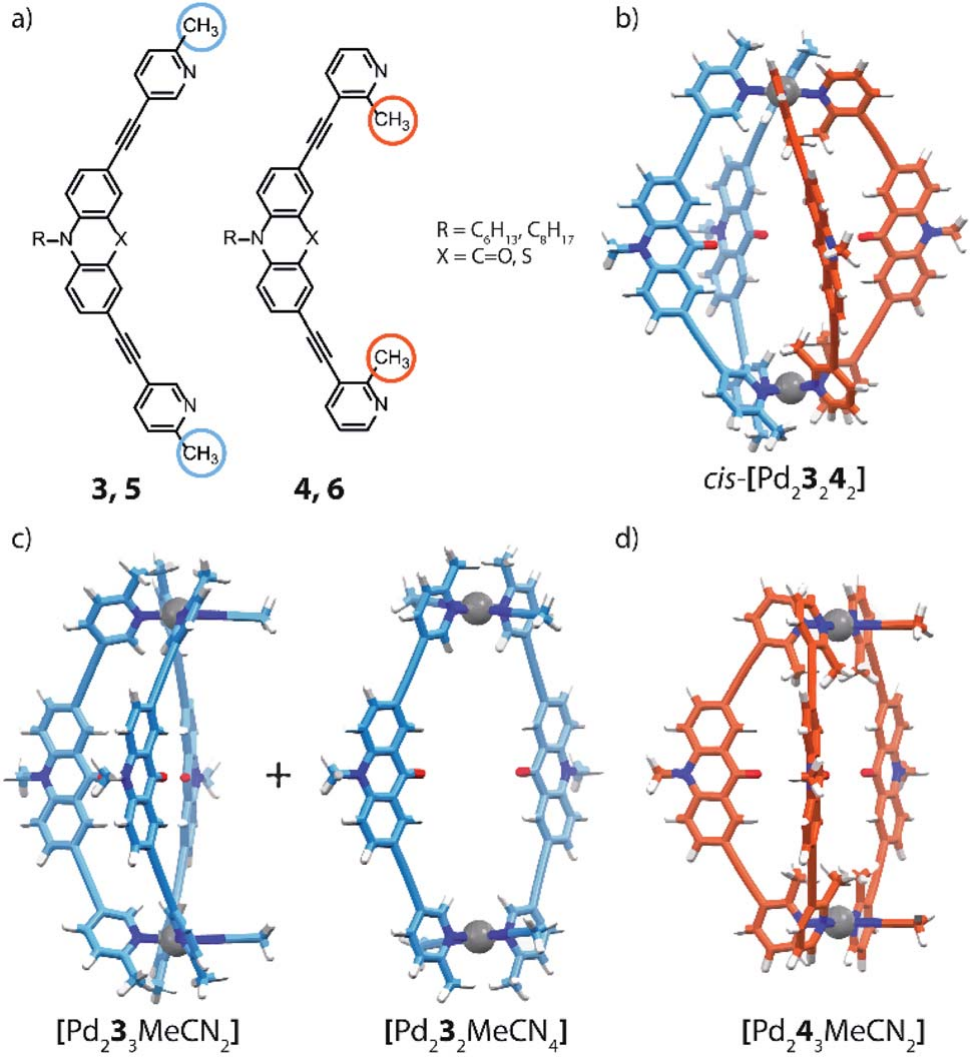
(a) Banana-shaped ligands with outside- and inside-pointing methyl groups; (b) DFT model of heteroleptic cage *cis*-[Pd_2_**3**_2_**4**_2_]; (c) DFT model of bowl and ring obtained with ligand **3** and (d) bowl obtained with ligand **4**.

The coordination sphere engineering strategy has recently been used by Clever and co-workers to assemble a heteroleptic *cis*-[Pd_2_L^A^_2_L^B^_2_] cage from bis-monodentate picolyl ligands.^66^ The authors reported four different ligand derivatives, were the picoline donor groups are connected to either acridone (**3**, **4**) or phenothiazine (**5**, **6**) backbones *via* their *meta* positions. Using either 5- or 3-substituted picolines resulted in an “inside” or “outside” orientation of the methyl groups with respect to the enclosed cavity upon cage formation. Steric hindrance around the coordination sites was found to disfavour the formation of homoleptic [Pd_2_**L**_4_] cages (except for a special case where anion templation triggers catenation to give a cage dimer, see below). By combining Pd^II^ ions with only a single acridone or phenothiazine ligand bearing the outside-pointing methyl group (**3**, **5**), a mixture of a [Pd_2_L_2_Solv_4_] ring and a [Pd_2_L_3_Solv_2_] bowl was formed. Pd^II^ coordination spheres were found to be saturated by coordinating solvent molecules (Fig. 5c). When using the ligands with inward-pointing methyl groups (**4**, **6**), a [Pd_2_L_3_Solv_2_] bowl is formed exclusively, suggesting a less steric hindrance than found among the outward-pointing picolyl donors (Fig. 5d).

The selective assembly of homoleptic [Pd_2_L_3_Solv_2_] bowls has been confirmed by means of 1D and 2D NMR spectroscopy, including ^1^H–^1^H NOESY experiments, which clearly indicate cross peaks between ligands occupying different positions in the bowl. In addition, DOSY-NMR, ESI-MS analysis as well as DFT modelling further supported the assignment of these structures. Comparison of Pd···Pd distances in the calculated structures shows the effect of steric hindrance resulting in contraction (methyl-in) or elongation (methyl-out) of the respective species. The reaction of **3** with **4**, bearing different methyl positions but similar backbones, with Pd^II^ cations in a 1 : 1 : 1 ratio, results in the formation of a new species that has been identified as *cis*-[Pd_2_**3**_2_**4**_2_]. Alkyl chains of different lengths were attached to the isomeric ligands to unambiguously identify the resulting heteroleptic compound by ESI-MS analysis. Additionally, ^1^H–^1^H NOESY cross peaks between the two different ligands provide confirmation for the heteroleptic structure. DFT calculations and the X-ray structure of a mononuclear model compound allowed to determine the *cis*-isomer of [Pd_2_**3**_2_**4**_2_] as more stable than the tentative *trans*-isomer. Finally, the authors investigated the coordination sphere engineering approach by combining two different backbones. Self-assembly of Pd^II^ with a 1 : 1 combination of the methyl-out acridone ligand and the methyl-in phenothiazine bridge resulted in the clear formation of a [Pd_2_L^A^_2_L^B^_2_] heteroleptic cage. Interestingly, when testing the inverted combination, NMR analysis revealed multiple overlapping proton signals, partially assigned to the acridone-based homoleptic bowl. Moreover, single crystals have been obtained from this mixture, revealing the surprising presence of an interpenetrated [Pd_4_**5**_8_] homoleptic double-cage (not shown), consisting of eight methyl-out phenothiazine-based ligands that show strong tilting and significantly distorted Pd^II^-coordination spheres. The driving force leading to this sterically overcrowded product is an anion templating effect, as has been reported before.^45^

**Fig. 6:**
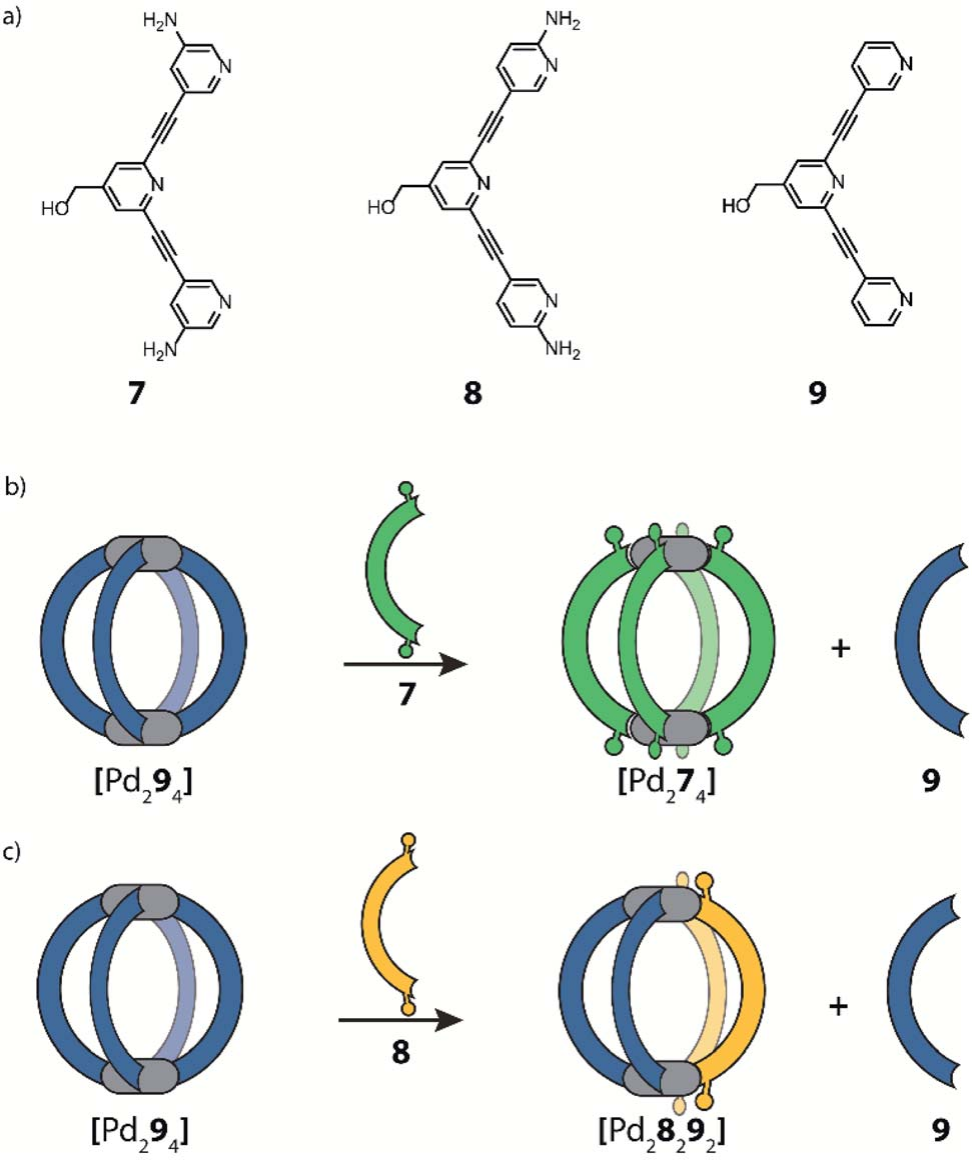
(a) Banana-shaped ligands bearing amino substituents at the pyridyl donor groups; (b) addition of ligand **7** to [Pd_2_**9**_2_] leads to complete displacement of **9**; (c) formation of heteroleptic cage *cis*-[Pd_2_**8**_2_**9**_2_] can be achieved by partial ligand displacement under kinetic control.

Instead of steric hindrance on the donor group, functionality that enables secondary weak interactions can be introduced. Crowley and co-workers reported a system where they take advantage of hydrogen bonding to stabilize a *cis*-[Pd_2_L^A^_2_L^B^_2_] heteroleptic cage.^67,68^ In these studies, amine substituents were installed on tris-pyridyl banana-shaped ligands, resulting in a 3-amino (**7**), 2-amino (**8**) or an unfunctionalized ligand (**9**). After formation of homoleptic cage [Pd_2_**9**_4_], functionalized ligands (**7** or **8**), both of which have stronger donor capacities than **9**, were added and ligand exchange results were monitored. By adding *meta*-substituted ligand **7**, bridges **9** of the homoleptic cage are completely displaced, transforming the system into homoleptic cage [Pd_2_**7**_4_] (Fig. 6b). The same experiment, using **8** instead, leads to a drastically different result. Here, the formation of a [Pd_2_**8**_2_**9**_2_] heteroleptic cage was observed, accompanied by the release of 2 equivalents of **9** (Fig. 6c). A combination of NMR analyses and DFT calculations suggested the formation of the *cis*-isomer, stabilized by the presence of hydrogen bonding between two neighbouring ligands **8**. Such stabilizing hydrogen bonds cannot form in the imaginable *trans*-isomer. Interestingly, this heteroleptic cage can be obtained only *via* the ligand displaced pathway. Attempts to form *cis*-[Pd_2_**8**_2_**9**_2_] directly from a combination of the building blocks resulted in a convoluted mixture of products, including [Pd_2_**8**_2_**9**_2_], while no homoleptic species were detected. Moreover, the authors reported that the heteroleptic cage gradually undergoes further ligand displacement until the formation of homoleptic [Pd_2_**8**_2_] is achieved. These observations, together with DFT energy calculations, point to the fact that the herein obtained heteroleptic compound is a kinetic product.

**Fig. 7:**
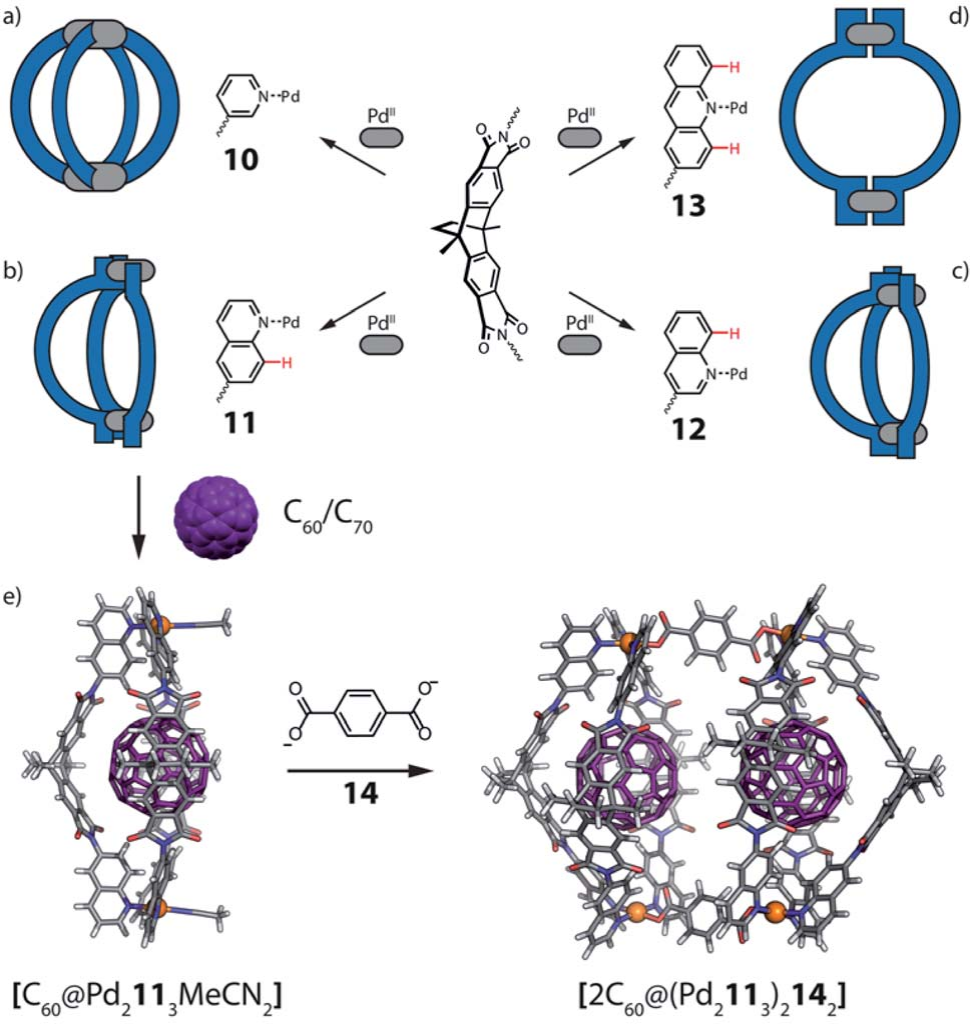
Formation of (a) cage [Pd_2_**L**_4_] when using pyridine donors; (b and c) [Pd_2_L_3_Solv_2_] bowls when using quinoline donors and (d) a [Pd_2_L_2_Solv_4_] ring using acridine donors. (e) Crystal structure of C_60_@[Pd_2_**11**_3_] bowl and DFT model of heteroleptic, pill-shaped dimer, binding two fullerenes C_60_.

Another approach of coordination sphere engineering, aimed at increasing structural complexity, has been developed by Clever and co-workers.^69,70^ In this strategy, steric hindrance is introduced around the metal coordination environment by using quinoline-based ligands instead of common pyridine derivatives. The authors designed a series of banana-shaped ligands bearing pyridines (**10**), two different quinoline isomers (**11**, **12**) or acridine (**13**) as donor groups (Fig. 7). The latter three cases feature hydrogen substituents in the annelated benzene rings that introduce steric pressure below/above the square-planar Pd^II^ coordination environment (Fig. 7b–d). Self-assembly of these ligands with Pd^II^ results in the formation of [Pd_2_**L**_4_] cages with pyridines, [Pd_2_L_3_Solv_2_] bowl-shaped structures with quinolines and [Pd_2_L_2_Solv_4_] rings when using acridines. All species were characterized by 1D and 2D NMR, high resolution ESI-MS and single crystal X-ray diffraction analysis. Furthermore, owing to the bent shape and π-surface of the ligand, both the cage and the bowl species were shown to bind C_60_ and C_70_ fullerenes in polar solvents. Interestingly, pyridyl-based cage [Pd_2_**10**_4_] can only bind C_60_ selectively, while the 6-quinolinyl-modified ligand **11** (with nitrogen donor on the outer ring and steric congestion on the inner benzene), allows to form a [Pd_2_**11**_3_Solv_2_] bowl with increased cavity dimensions as well as open geometry, able to encapsulate also the bigger C_70_. Owing to the bowl geometry, bound C_60_ exposes about 25% of its surface area to the solution environment. This allowed the authors to perform a Diels–Alder reaction on the bound fullerene, leading to the selective formation of only the mono-adduct. Interestingly, attempts to crystallize the empty bowl species resulted in single crystals of a [Pd_2_**11**_4_] cage, not observed in solution, suggesting a fine energetic balance between the two species.

When reacting a 3-quinolinyl modified ligand (**12**) with Pd^II^ cations at room temperature, the obtained compound is again a [Pd_2_**12**_3_Solv_2_] bowl. However, compared with the analogous bowl formed from **11**, it is unstable at room temperature, partially converting to a [Pd_2_**12**_4_] cage over two days. Again, cage formation has been further confirmed by single crystal X-ray diffraction analysis. A DFT comparison of the species' relative stability in the cage/bowl equilibria for the quinoline-based ligands **11** and **12** supports the experimental data, showing that the bowl-shaped compound is favoured when the steric pressure is in the inward-pointing benzene ring (as in **11**), while the cage compound is favoured when the steric pressure is moved to the outer part of the ligand (as in **12**). Surprisingly, the cage formed using **12** is unable to bind any fullerene derivative, probably due to its more twisted shape and hence smaller cavity.^70^

As shown before, introduction of steric pressure around the metal ion can prevent saturation of the coordination sphere with four nitrogen donor ligands. As remaining coordination sites cannot be left unoccupied on Pd^II^ cations, they are usually filled by solvent molecules or small ligands such as halide anions (F^−^, Cl^−^, Br^−^) or others. Therefore, bowl and ring assemblies can act as platform for the addition of a further ligand leading to more complex heteroleptic species. Using the [Pd_2_L_3_(CH_3_CN)_2_] bowl-shaped assembly, Clever *et al.*^69^ were able to substitute the solvent molecules with di-carboxylate bridges. Addition of two terephthalate anions **14** to the preformed bowl species [Pd_2_**11**_3_] allowed to further increase the structural complexity of the system and form a unique heteroleptic pill-shaped assembly (Fig. 7e). This approach could further be extended from the empty bowls to the host–guest systems, thus affording a cavity harbouring two fullerenes (C_60_ or C_70_) in close vicinity.

Another example of combining different donors, namely pyridines and carboxylates, to form heteroleptic architectures has been explored by Stang and co-workers.^52^ A series of 2D and 3D compounds was reported by assembling *cis*-protected Pd^II^ fragments with terephthalate bridges and pyridine-based ligands. Balancing charges on the metal centres, deriving from the neutral and anionic donor groups, drive the formation of heteroleptic compounds by simply mixing these building blocks in the correct stoichiometric ratio. Linear bis-pyridine ligands lead to 2D metallocycles while tris- or tetra-pyridine ligands lead to 3D trigonal and tetragonal prisms, respectively. In summary, coordination sphere engineering (CSE) has been established as a reliable tool to synthesize either heteroleptic coordination cages directly or unsaturated bowl and ring species that serve as platforms toward the formation of higher order heteroleptic structures (see also Fig. 27). To reach non-statistical assembly, either homoleptic structures are disfavoured by repulsive steric or strain effects, or formation of heteroleptic species is promoted by attractive secondary interactions or charge balancing effects (and related *trans*-influences) in immediate vicinity of the coordinated metals. In the following, we move focus from the coordination spheres to the overall structure of the entire architectures, thus categorizing the subsequent approaches as “assembly-based” ones.

### 2.3 Shape complementarity

Design and assembly of coordination-driven supramolecular compounds is often realized using geometrical principles.^3,4,71^ The combination of the high directionality of many transition metal coordination bonds with controllable geometry of polytopic ligands allows to assemble a plethora of highly symmetric compounds based on the so called “edge-directed self-assembly” principle.^20^ These concepts have been used with kinetically rather inert, non-dynamic systems to develop heteroleptic compounds. For example, the use of organometallic fragments can be a valuable tool to synthesize supramolecular compounds bearing multiple ligands and functions in a stepwise manner. When using “naked” metal ions such as Pd^II^ in Werner-type complexes, building blocks are usually allowed to exchange dynamically, resulting in a less controllable situation when more than one type of ligand is present. The shape complementarity approach (SCA) takes advantage of geometric principles to selectively obtain one specific product by avoiding the formation of statistical mixtures containing other ligand combinations. Therefore, ligand angles are designed in order to precisely fit each other between matching bridges (often pairwise), resulting in enthalpic destabilization of strained homoleptic species, as well as overall entropic gain if the heteroleptic product has a lower nuclearity compared to one or more of the homoleptic precursors.

**Fig. 8:**
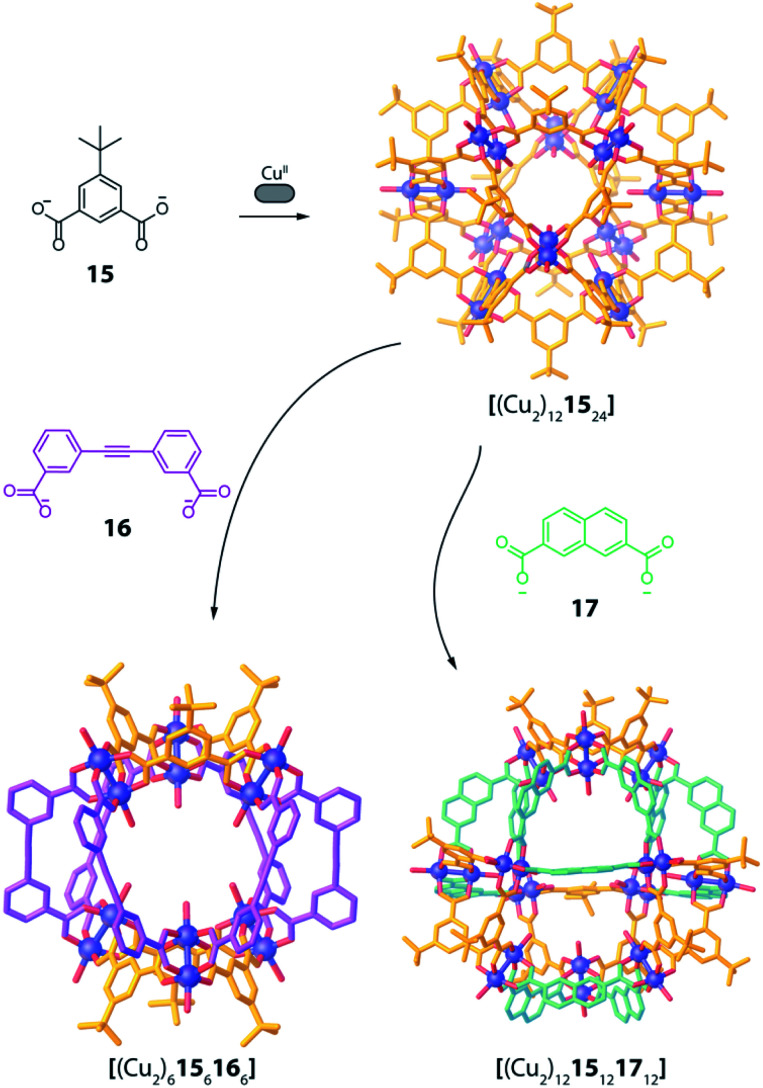
Ligand substitution transforms homoleptic rhombic cuboctahedron [(Cu_2_)_12_**15**_24_] into heteroleptic cages [(Cu_2_)_6_**15**_6_**16**_6_] and [(Cu_2_)_12_**15**_12_**17**_12_] when using bent dicarboxylate ligands.

This approached has been successfully used by Zhou and co-workers in 2010 in a series of Cu^II^-based cages with ditopic carboxylate ligands featuring different binding angles.^72^ The authors examined diverse ligands, binding pairs of Cu^II^ ions in “paddle-wheel mode” with local C_4_ symmetry, to form several homoleptic compounds. Among all reported architectures, one homoleptic cage was found to transform into heteroleptic cages by ligand substitution. In particular, Cu^II^ ions self-assemble with 5 *t*-butyl-1,3-benzenedicarboxylate (**15**), possessing a 120° binding angle, to form a [(Cu_2_)_12_**15**_24_] homoleptic cage of rhombic cuboctahedral geometry (Fig. 8). Reacting this cage with a larger 3,3′-(ethyne-1,2-diyl)dibenzoate ligand **16**, having a 60° binding angle, resulted in the formation of a [(Cu_2_)_6_**15**_6_**16**_6_] heteroleptic cage with lower nuclearity, suggesting an entropic stabilization of the final product (Fig. 8). Interestingly, when reacting the homoleptic architecture with 2,7-naphthalene dicarboxylate (**17**), having the same 120° binding angle but longer length, another heteroleptic cage is formed *via* ligand substitution. Different from the previous case, the compound's nuclearity is preserved ([(Cu_2_)_12_**15**_12_**17**_12_]), suggesting enthalpic contributions to govern stabilization of the product rather than entropic ones. Formation of all three species has been confirmed primarily by single crystal X-ray analysis, therefore the role of solid-state packing effects cannot be ruled out to significantly contribute to stabilizing the observed heteroleptic compounds.

**Fig. 9:**
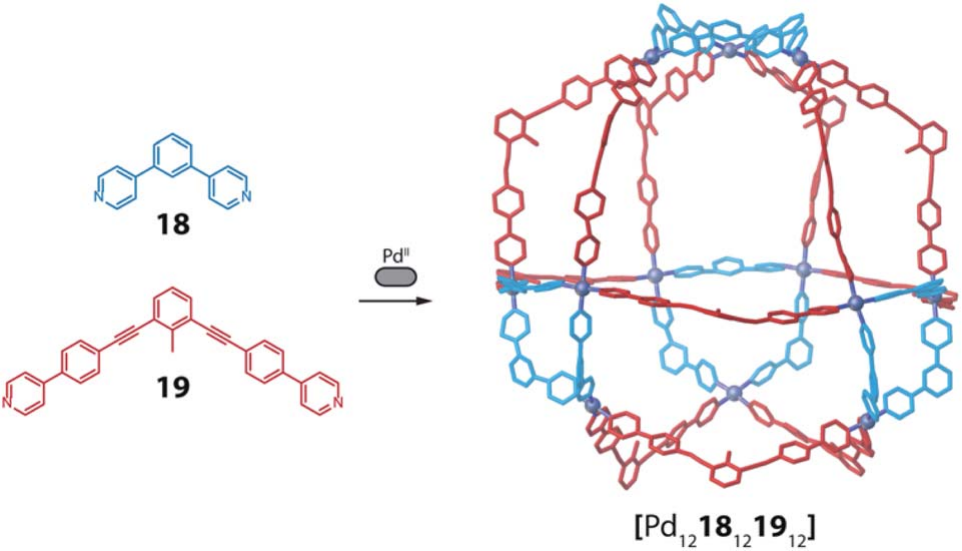
Self-assembly of a heteroleptic cantellated tetrahedron by combining two bis-pyridine ligands with Pd^II^ ions.

A similar approach was developed by Fujita and co-workers to synthesize a heteroleptic [Pd_12_L^A^_12_L^B^_12_] architecture by self-assembly of Pd^II^ cations with banana-shaped, pyridine-based ligands.^73^ The authors elucidated the geometric criteria to form large, homoleptic [Pd_*n*_L_2*n*_] Archimedean assemblies, leading to a [Pd_12_L_24_] cuboctahedron,^74^ a [Pd_24_L_48_] rhombicuboctahedron,^32^ up to a [Pd_30_L_60_] icosidodecahedron,^75^ by tuning the ligands' binding angles, lengths and flexibility. In order to further develop this principle to achieve heteroleptic assembly, two bis-pyridine ligands (**18**, **19**) bearing the same binding angle of 120°, but different length, were employed. Combining each of these ligands alone with Pd^II^ cations leads to the formation of homoleptic [Pd_12_L_24_] spheres. Self-assembly of both ligands with the metal centre in a 1 : 1 : 1 ratio, however, resulted in clean integrative self-sorting to give a [Pd_12_**18**_12_**19**_12_] heteroleptic structure in the shape of a “cantellated tetrahedron” (Fig. 9).

Formation of the compound was confirmed by a combination of NMR and ESI-MS analysis with single crystal X-ray diffraction. The study has been further extended including more ligands with different angles and lengths. The ligand-length ratio plays a crucial role on driving the system towards the integrative self-sorting of one defined compound or to statistical mixtures of [Pd_12_L^A^_*n*_L^B^_24−*n*_] (*n* = 1–24) species, that contain up to 7 × 10^5^ possible ligand combinations (stoichiometries and stereoisomers). A variation of this system, exhibiting particular beauty, was obtained when a short ligand was covalently bridged by a ten-membered chain to the concave face of its longer sibling. Here, narcissistic self-sorting of the ligand sites was observed, forming a sphere-in-sphere complex as confirmed by DOSY NMR analysis and a crystal structure.^76^ Interestingly, a comparison of the heteroleptic spheres reported by Zhou and Fujita shows the presence of a triangular substructure in which three metal ions are connected through the short ligands. As these ring fragments are conserved both in Zhou's carboxylate-Cu-system, and also present in Fujita's pyridine-Pd-sphere, they seem to play an important role for the overall stability of the heteroleptic architectures.

**Fig. 10:**
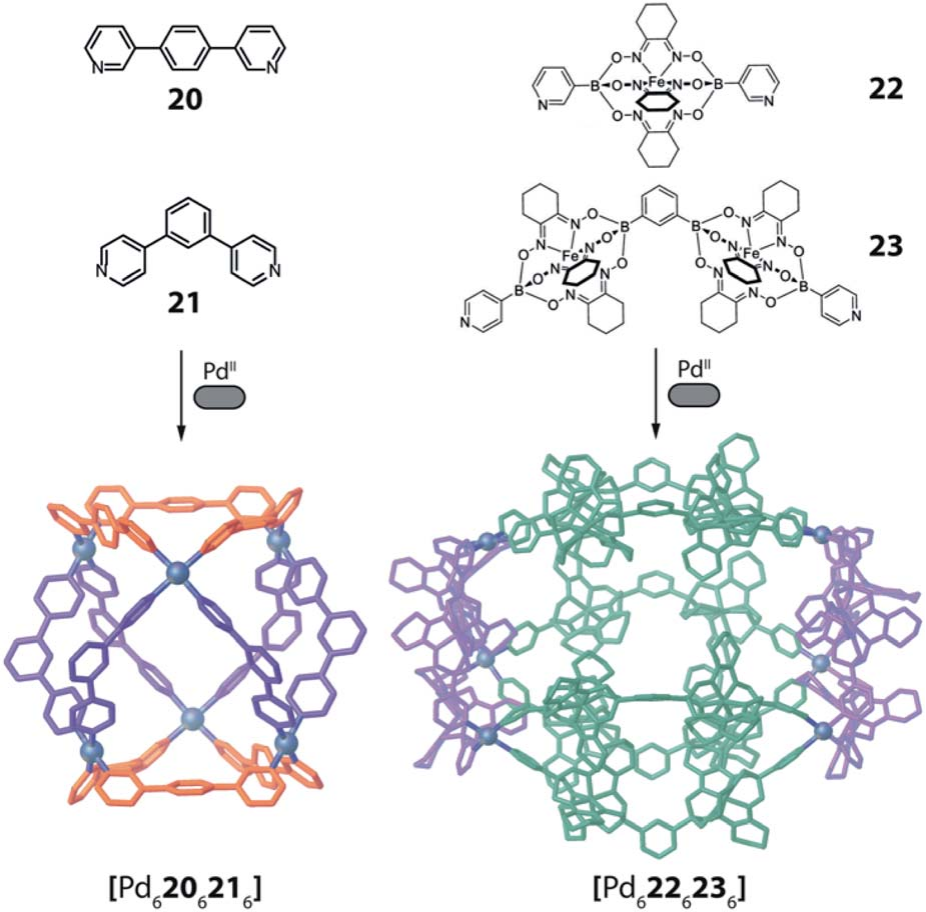
Two examples of barrel-shaped 
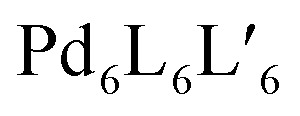
 assemblies from the combination of bis-monodentate ligand pairs with different donor bonding angles.

Severin and coworkers showed very recently that a topology similar to the one obtained by Zhou from carboxylate ligands and Cu^II^ cations in [(Cu_2_)_6_**15**_6_**16**_6_] can also be realized using a simple set of bis-pyridyl ligands **20** and **21** with Pd^II^ cations (Fig. 10 left).^77^ Interestingly, the compound was selectively formed from a mixture of six bis-pyridyl ligands with different lengths and donor angles, thus highlighting its extraordinary stability. Furthermore, the same topology could also be achieved with a set of much more elaborate ligands **22** and **23** based on the iron-clathrochelate chemistry (Fig. 10 right).

Clever and co-workers reported an alternative approach to achieve integrative self-sorting of heteroleptic cages using shape complementarity. Geometric design, with complementary binding angles, was used here to explore the formation of rather small [Pd_2_L^A^_2_L^B^_2_] cages. The first study has been conducted using three banana-shaped ligands: an inward-bent acridone-based bridge having *iso*-quinoline donor groups (**25**), an outward-bent phenanthrene-based ligand (**24**) as well as carbazole-based ligand **26**, both having pyridine donors, with binding angles of 60°, 120° and 75° respectively.^78,79^ Each of the three ligands mixed with Pd^II^ cations individually forms homoleptic species. From **25**, a [Pd_2_**25**_4_] cage is formed in which the *iso*-quinoline donors induce a heavy distortion, forcing the assembly into a strongly twisted, presumingly high energy conformation. The use of **24**, due to its shorter length and larger binding angle, leads to the formation of a tetranuclear [Pd_4_**24**_8_] ring. Self-assembly of Pd^II^ with the carbazole-based **26** result in a lantern-shaped [Pd_2_**26**_4_] cage which was previously shown to give an interpenetrated [Pd_4_**26**_8_] double cage upon addition of chloride anions as templates.^80^

**Fig. 11:**
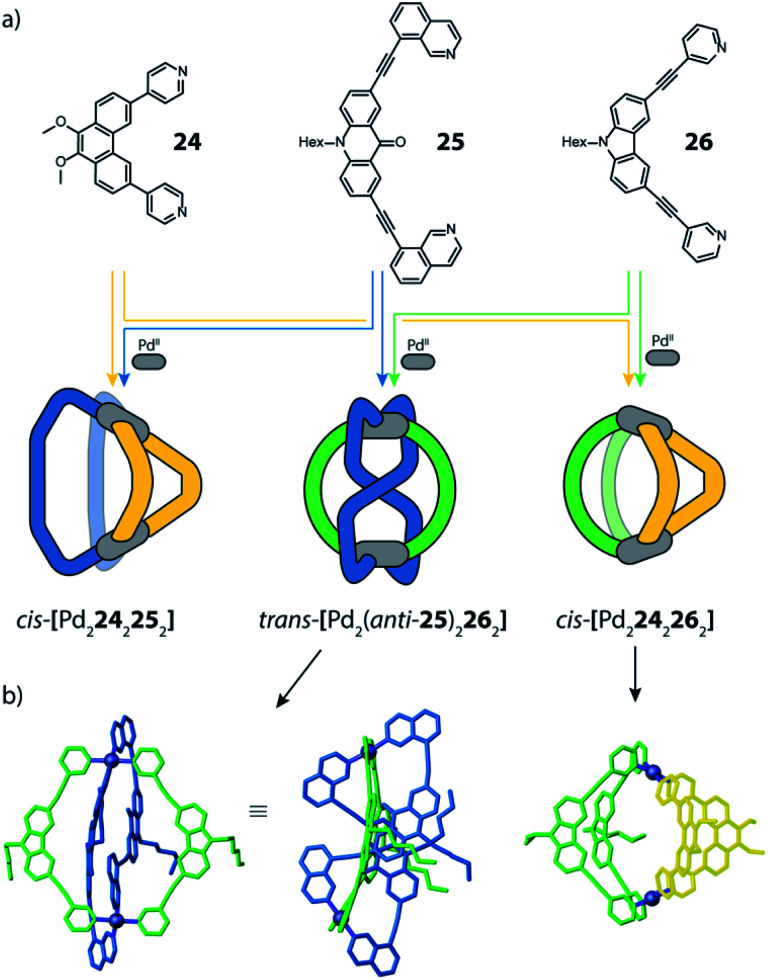
(a) Self-assembly of *cis*- and *trans*-heteroleptic cages using shape complementarity; (b) single crystal X-ray structures of *trans*-[Pd_2_(*anti*-**25**)_2_**26**_2_] and *cis*-[Pd_2_**24**_2_**26**_2_].

The first two ligands possess complementary binding angles of 60° and 120°, respectively, which gave rise to the prediction of a *cis*-[Pd_2_L^A^_2_L^B^_2_] heteroleptic cage, as supported by DFT calculations. Indeed, the combination of these two ligands with a Pd^II^ source in a 1 : 1 : 1 stoichiometric ratio leads to the clean formation of the desired cage *cis*-[Pd_2_**24**_2_**25**_2_], as confirmed by NMR and ESI-MS analysis (Fig. 11a). Additionally, ^1^H–^1^H NOESY analysis clearly shows cross peaks consistent with the *cis* stereochemistry. The authors further tested the shape complementarity of ligands **24** and **25**, each, with carbazole-based ligand **26**. Combination of **24**, **26** and Pd^II^ in a 1 : 1 : 1 ratio also led to the clean formation of a *cis*-configured cage [Pd_2_**24**_2_**26**_2_], even though in this case, the binding angles of 75° and 60° are not really matching (Fig. 11a). Formation of the *cis*-cage was again confirmed by NMR and ESI-MS analysis, as well as *via* single crystal X-ray diffraction (Fig. 11b). Self-assembly of carbazole-based ligand **26** with acridone-based ligand **25** also leads to the exclusive formation of a [Pd_2_L^A^_2_L^B^_2_] species, as revealed by prominent peaks in the high-resolution ESI-MS spectra. However, in this case, ^1^H-NMR analysis resulted in a very complicated spectrum, with ligand signals split due to significant desymmetrization effects. The riddle could be solved through single crystal X-ray analysis, revealing a new structural motif for self-assembled coordination cages: a *trans*-[Pd_2_(*anti*-**25**)_2_**26**_2_]_4_ assembly in which both acridone ligands assume an *anti*-conformation instead of the common *syn*-conformation resulting in a “figure-eight”-shaped (Pd_2_**25**_2_)-substructure flanked by two ligands **26**. In other words, the system adopts a self-penetrating, chiral topology in which both acridone ligands pass through the middle of the assembly, thus sacrificing the inner cavity (Fig. 11b). Interpretation of the NOESY cross-peaks allowed to confirm this configuration also in solution. Interestingly, due to the dynamic behaviour of the Pd^II^-pyridine/quinoline coordination bonds, all three heteroleptic cages can be achieved also by mixing the corresponding homoleptic assembly pairs in the correct stoichiometric ratio, thus triggering a cage-to-cage transformation process. This clearly indicates that for all three reported cases, the heteroleptic compound is the thermodynamic product. Explanation for the species involving the acridone-based ligand is certainly found in its highly distorted and strained homoleptic assembly, giving rise to enthalpic drive to react with shape complementary counter ligands to give the structurally relaxed heteroleptic products. At the same time, when using the phenanthrene-based ligand, the entropic contribution deriving from the transformation of tetranuclear [Pd_4_L_8_] rings into a multitude of [Pd_2_**L**_4_] cages, is non-neglectable. Finally, the authors could prove higher stability of and better structural control over the *cis*-[Pd_2_**24**_2_**25**_2_] cage, with both ligands showing perfectly complementary binding angles: addition of the missing ligand to one of the other two cages was found to lead to ligand exchange, replacing the carbazole component and resulting in a heteroleptic-to-heteroleptic cage transformation to give *cis*-[Pd_2_**24**_2_**25**_2_].^79^

**Fig. 12:**
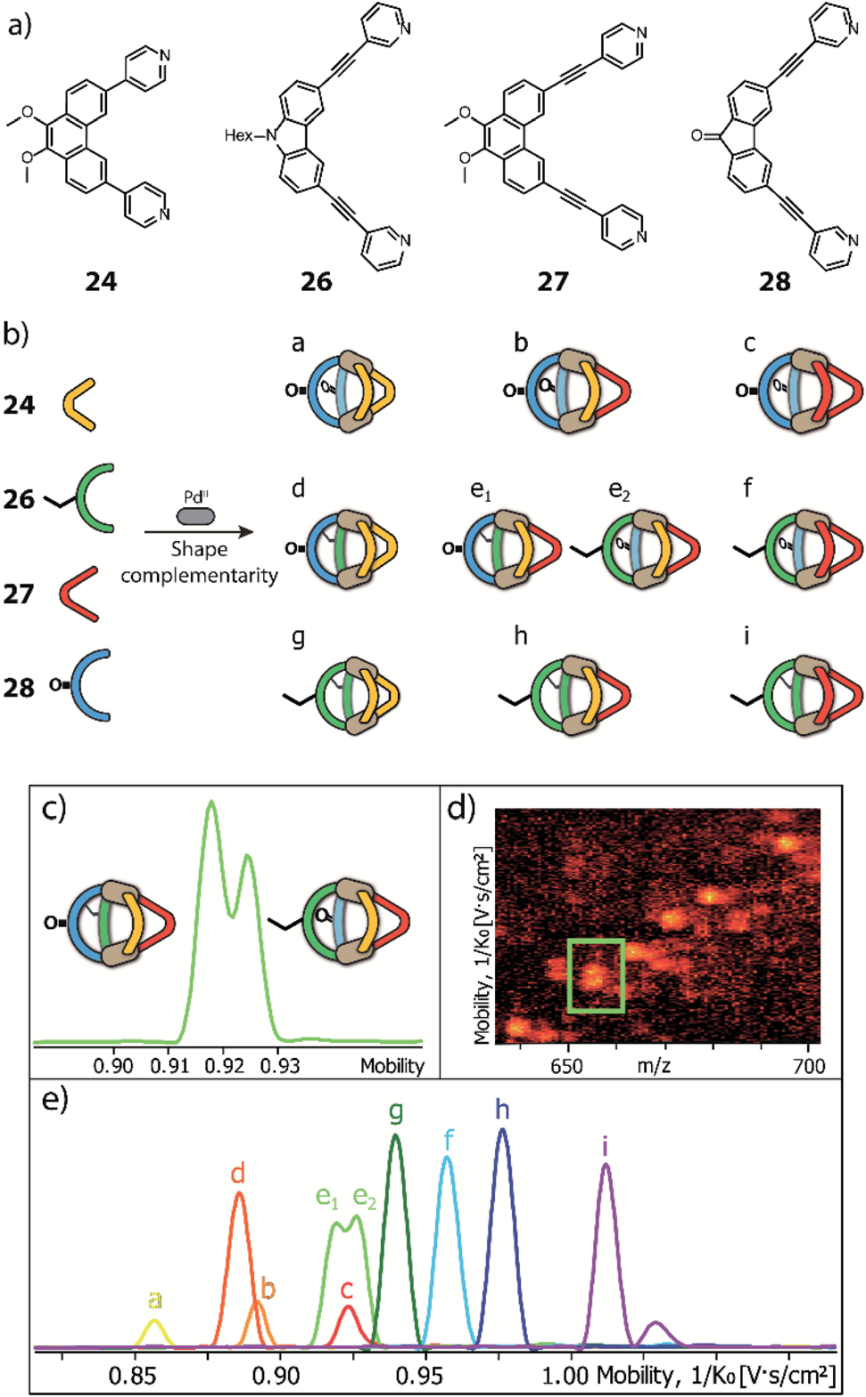
(a) Two pairs of shape-complementary banana-shaped bis-pyridine ligands; (b) ten possible heteroleptic combinations obtained by mixing the four ligands to form [Pd_2_**L**_4_] cages, directed by shape complementarity; (c) high resolution mobilogram showing differentiation of the two possible [Pd_2_L^A^L^B^L^C^L^D^] isomers; (d) partial heat map and (e) full mobilogram of all ten cages. Reproduced from ref. 81 with permission from the Royal Society of Chemistry, copyright 2019.

To further study the versatility of the shape complementarity approach, Clever and co-workers increased the degree of complexity in the system.^81^ The combination of three or four shape complementary ligands resulted in statistical formation of species that still obey a certain degree of self-sorting based on shape complementarity of the concave/convex ligand groups in [Pd_2_L^A^_2_L^B^_*x*_L^C^_2−*x*_] (*x* = {0–2}) and [Pd_2_L^A^_*x*_L^B^_2−*x*_L^C^_*y*_L^D^_2−*y*_] (*x* = {0–2}; *y* = {0–2}) (Fig. 12). Such systems are challenging, if not impossible, to be analyzed by common NMR techniques, including COSY and DOSY spectra, therefore ESI-MS combined with Trapped Ion Mobility Spectrometry (TIMS) was employed, a technique that has just recently found entry in the analysis of supramolecular systems.^82^ The combined analysis of experimentally determined and theoretically computed collisional cross sections (CCS) then allowed to differentiate the constituents of the mixture. For this study, two new ligands were added to the previously reported phenanthrene- and carbazole-based ligands (**24**, **26**). An elongated phenanthrene-based ligand (**27**), where the pyridine donor group is connected to the backbone through an alkyne bridge, still having 60° binding angle, and a fluorenone-based ligand (**28**) with binding angle (80°) and size comparable with the carbazole one. The set of four building blocks can be divided in two “long” ligands (**26**, **28**) and two “short” ligands (**24**, **27**).

Firstly, shape complementarity has then been tested among the four possible long–short combinations, resulting in the clean formation of *cis*-[Pd_2_L^A^_2_L^B^_2_] cages in all cases, as confirmed by NMR and ESI-MS analysis (Fig. 12). Then, the ternary systems made by one long ligand and the two short phenanthrenes were investigated, resulting in a distribution of [Pd_2_L^A^_2_L^B^_2_], [Pd_2_L^A^_2_L^B^L^C^] and [Pd_2_L^A^_2_L^C^_2_] cages. The analysis of already such a mixture proved challenging, as NMR suffered from extensive signal overlap and symmetry-related signal splitting. Due to the small structural differences among the three cages, also DOSY-NMR failed to provide a clear differentiation. Instead, using TIMS analysis allowed to successfully distinguish all different constituents in the system by obtaining separate ion mobility values. Subsequently, this analytical tool was applied to a mixture of all four ligands. While the number of possible species (ten) is already reduced from 55 that would result from full statistical distribution thanks to the shape complementarity approach, the analysis would not have been possible with NMR methods. Of the ten contained species, nine possess different ligand combinations (thus also differing in their masses), but the cage comprising all four ligands can be present as both a *cis*- and a *trans*-[Pd_2_L^A^L^B^L^C^L^D^] isomer, giving rise to the same ESI-MS signature. Remarkably, ion mobility measurements allowed to differentiate all ten species, including the *cis*- and *trans*-isomeric species containing all four ligands, despite their small differences in the collisional cross sections. In addition, all signals have then been unambiguously assigned with the help of theoretical CCS value calculations.

In conclusion, shape complementary assembly (SCA) has been proven to be a versatile approach for accessing heteroleptic structures, owing to a combined action of enthalpic and entropic effects. Architectures featuring different nuclearity have been reported and both large and smaller structures can be achieved in this way.

### 2.4 Non-symmetric ligands

The approach discussed now relies on the use of non-symmetric bis-monodentate ligands, either by changing the donor groups or introducing asymmetry in the ligand geometry. It is worth to mention that non-symmetric ligands can be designed to form heterometallic supramolecular cages, showing structural complexity and even multifunctional properties, as reported in the literature.^26,83–89^ However, most of the reported examples still give rise to rather symmetric compounds and therefore will not be treated in this review. Nevertheless, the introduction of different metal centres it is an interesting tool to develop multifunctional, coordination-driven systems.

Recently, some examples of using non-symmetric ligands to increase the structural complexity of homometallic architectures have emerged. Combined with other approaches, such as the above-described coordination sphere engineering and shape complementarity, breaking the ligand symmetry will allow powerful ways to tailor complex multifunctional materials in the future. It is worth noting, however, that most so far reported systems rely on only one type of ligand per assembly and while individual metal nodes may be heteroleptic, the resulting entire assemblies are in many cases considered to be homoleptic species.

**Fig. 13:**
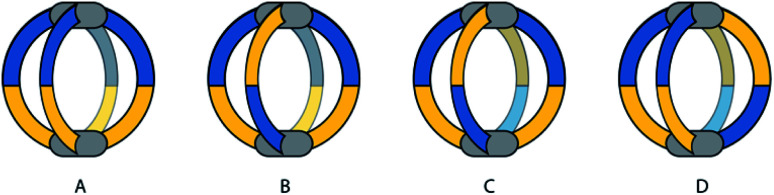
Four possible isomers of [M_2_L_4_] cages using non-symmetric ligands, namely (A) “four up”, (B) “three up, one down”, (C) and (D) *trans*- and *cis*- “two up, two down”.

**Fig. 14:**
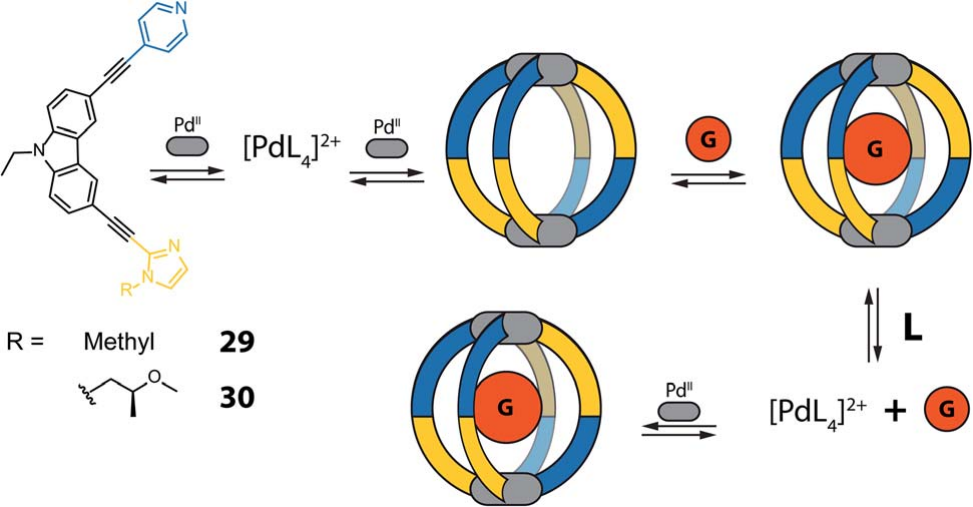
Self-assembly and guest binding of *cis*-[Pd_2_**L**_4_] cages composed of non-symmetric ligands with two different donor groups. Controlling the metal to ligand ratio allows to assemble/disassemble the cage and affect guest binding.

Yuasa and co-workers recently reported a system based on ditopic banana-shaped carbazole ligands, bearing two different donor groups: *para*-substituted pyridine and imidazole. Ligand **29** comes with a simple alkyl substituent on the imidazole and derivative **30** carries a chiral substituent in the same position (Fig. 14).^90^ Both ligands self-assemble with Pd^II^ ions to give [M_2_L_4_] cages. When using such non-symmetric ligands, four possible [M_2_L_4_] isomers can be proposed (Fig. 13). Having two different donor groups resulted in a two-step self-assembly mechanism as shown *via* UV-Vis titrations. Moreover, the authors could isolate a mononuclear [Pd**29**_4_] compound, under careful stoichiometric control of the metal-to-ligand ratio, proving with a combination of NMR analysis and DFT calculations that in this case only the imidazole donors coordinate the Pd^II^ ions. Despite the possibility to obtain four different isomers, only the one having two pyridine and two imidazole donor groups on every Pd^II^ ion in a *cis*-arrangement was obtained (Fig. 13, isomer D). Introduction of a chiral substituent on ligand **30** resulted in formation of two diastereomers, with NMR signal splitting for the protons of imidazoles and pyridines. Finally, the authors studied the guest binding properties of the system. Addition of ReO_4_^−^ caused the formation of a host–guest complex with cage [Pd_2_**30**_4_], while it did not bind to the [Pd**30**_4_] mononuclear species (Fig. 14). After addition of two extra equivalents of ligand to [Pd_2_**30**_4_], the cage reorganizes to the mononuclear [Pd**30**_4_] complex and expels the guest, suggesting a highly dynamic system with potential to develop even more complex on/off guest binding switches.

Another example of using non-symmetric ligands to control and increase structural complexity of [M_2_L_4_] cages has been reported by Lewis and co-workers.^91^ The system design relies on bis-monodentate banana-shaped ligands with two different donors, featuring steric constraints, geometric complementarity or a combination of both effects. To explore the first effect, the authors designed a ligand having as donor group a pyridyl moiety on one side and a picolyl donor group on the other (**31**). Again, four possible isomers are expected when using such a non-symmetric ligand (Fig. 13). Reaction of **31** with Pd^II^ turned out to give a solvent dependent system. In acetonitrile only the *trans*-[Pd_2_**L**_4_] species is achieved (Fig. 13, isomer C), while in DMSO a mixture of *cis*- and *trans*-[Pd_2_**L**_4_] isomers is formed, as confirmed by NMR analysis and DFT calculations.

**Fig. 15:**
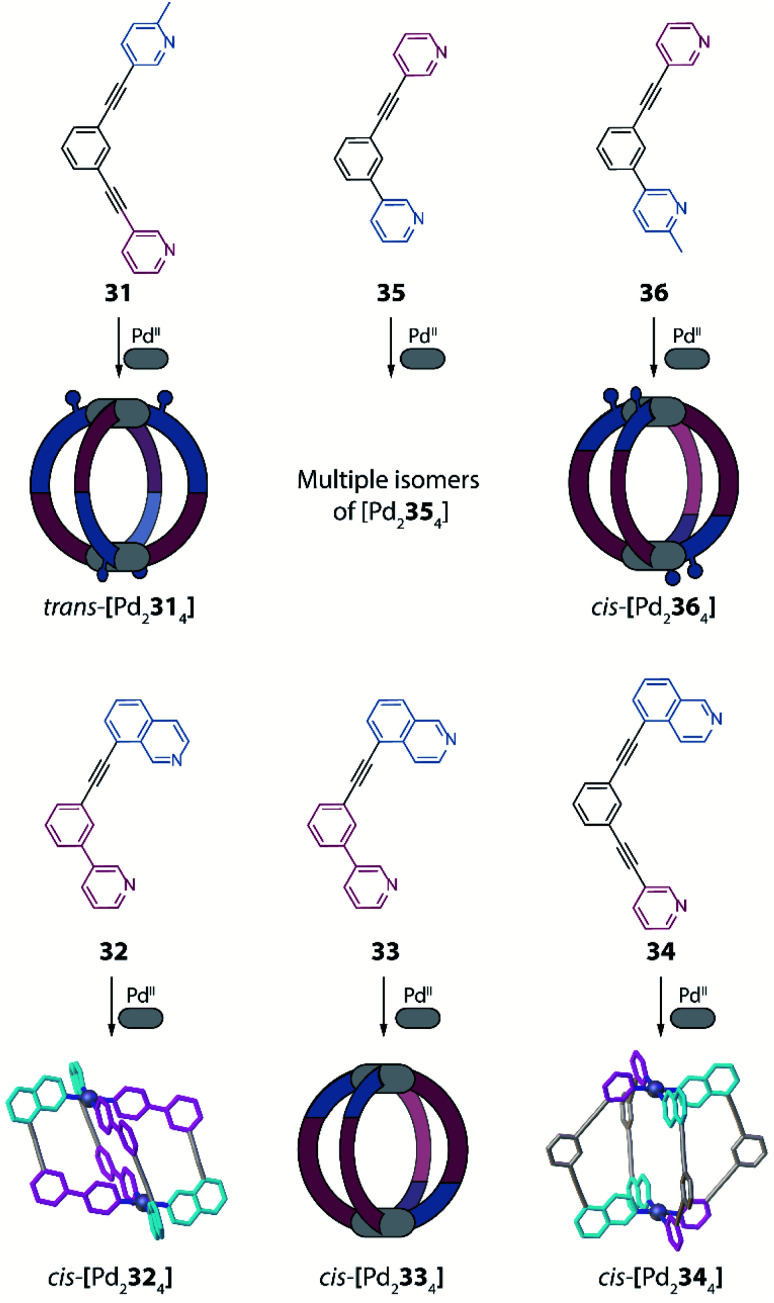
Combination of non-symmetric ligands with geometric complementarity and coordination sphere engineering to form a series of homoleptic cages with reduced symmetry.

Subsequently, the geometric complementarity within homoleptic cages deriving from non-symmetric ligands **32**, **33**, **34** and **35**, based on combinations of pyridyl and isoquinoline donor groups, was explored. For these ligands, the bonding vector of the two nitrogen donors is not parallel, therefore suggesting the formation of *cis*-[Pd_2_**L**_4_] cages, according to shape complementarity principles (Fig. 13, isomer D). Indeed, self-assembly of either **32**, **33** or **34** with Pd^II^ cations results in the formation of the expected *cis*-[Pd_2_**L**_4_] cages, as confirmed by a combination of NMR analyses, single crystal X-ray structures for *cis*-[Pd_2_**32**_4_] and *cis*-[Pd_2_**34**_4_], as well as DFT calculations for all isomers of [Pd_2_**33**_4_] (Fig. 15). Reaction of Pd^II^ cations with ligand **35** likewise resulted in the formation of species with stoichiometry [Pd_2_**35**_4_], however, in this case a complex NMR spectrum suggest the co-existence of more than one isomer. DFT calculations for the four possible isomers show that the energy difference between the *cis*-conformer and the conformer having three identical donor groups on the same side is negligible (0.1 kJ mol^−1^; Fig. 13, isomer B), resulting in a mixture of species. Finally, the authors explored the combination of both steric hindrance and geometric complementarity with ligand **36**. Introduction of a methyl substituent was found to change the energetic balance again towards *cis*-isomer *D*, resulting in formation of one main compound, identified as *cis*-[Pd_2_**36**_4_] by a combination of 2D NMR analysis and DTF calculations (Fig. 15). Recently, Lewis extended the principle by introducing different functional groups in exohedral positions *via* click chemistry.^92^

A nice example for the realization of a [Pd_2_**L**_4_] structure with an all-parallel arrangement of four non-symmetric bis-monodentate pyridyl bridges (Fig. 13, isomer A) was reported by Natarajan *et al.* based on the chiral steroid cholic acid.^93^

Another example has been recently reported by the group of Chand, where the self-assembly of naked and *cis*-protected Pd^II^ ions combined with a non-symmetric ligand having pyridine and aniline donor groups was investigated. Among all the possible assembly products, a combination of NMR and DFT studies proved the selective formation of a *cis*-[Pd_2_**L**_4_] structure with a “two up two down” arrangement of the non-symmetric ligand (Fig. 13, isomer D).^94^

### 2.5 Backbone-centred steric hindrance

**Fig. 16:**
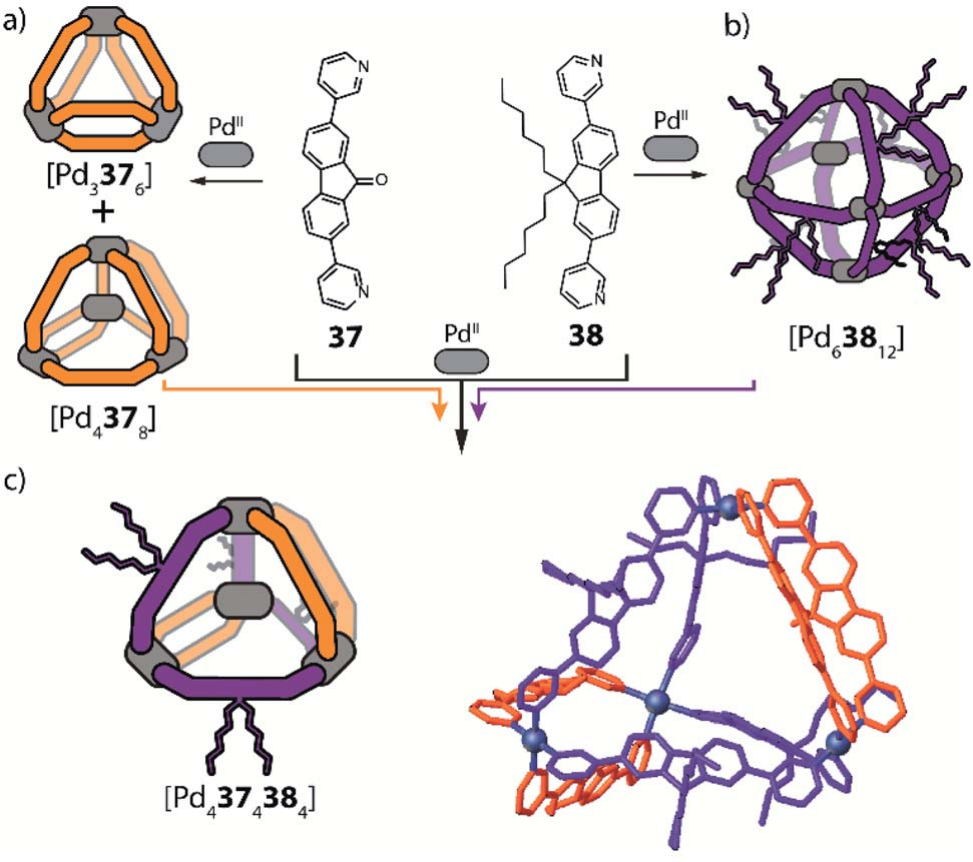
(a) Ligand **37** with a small backbone substituent forms a mixture of [Pd_*n*_L_2*n*_] species; (b) introduction of steric bulk selects exclusively a large [Pd_6_L_12_] octahedron; (c) steric hindrance combined with the presence of two non-equivalent edges in the tetrahedral topology leads to the formation of an unprecedented [Pd_4_L^A^_4_L^B^_4_] heteroleptic tetrahedron.

Recently, Clever and co-workers developed a new approach to achieve integrative self-sorting of unprecedented [Pd_4_L^A^_4_L^B^_4_] heteroleptic tetrahedra.^95^ The system is based on a flat, non-linear bridge **37**, that self-assembles with Pd^II^ cations to give a solvent-dependent dynamic library of species [Pd _*n*_**37**_2*n*_] (*n* = 3, 4, 6) (Fig. 16a). The assemblies adopt different topologies, a [Pd_3_**37**_6_] ring, a [Pd_4_**37**_8_] tetrahedron (similar to the ones described in Section 2.1) and a [Pd_6_**37**_12_] octahedron as minor component (not shown). Introduction of a bulkier substituent in ligand **38** resulted in the exclusive formation of a large [Pd_6_**38**_12_] octahedron (Fig. 16b). The combination of Pd^II^ with both ligands **37** and **38** (or mixing of cages [Pd_*n*_**37**_2*n*_] and [Pd_6_**38**_12_]) resulted in the exclusive formation of a [Pd_4_**37**_4_**38**_4_] heteroleptic tetrahedron (Fig. 16c), as confirmed by NMR spectroscopy, high resolution ESI-MS spectrometry, as well as a single crystal XRD structure. Reason for clean integrative self-sorting is the presence of two non-equivalent edges in the [Pd_4_L_8_] tetrahedral topology (Section 2.1), combined with the presence of steric bulk in the backbone of ligand **38**. Flat ligand **37**, featuring only a small substituent in its central backbone position, can occupy the two doubly bridged edges, having its carbonyl substituent pointing either inside the cage or to the π-surface of the neighbouring ligand backbone. On the other hand, the four single-spanned edges are occupied by **38**, with the bulky alkyl chains pointing outside and therefore escaping from steric pressure. Cage-to-cage transformation proved [Pd_4_**37**_4_**38**_4_] to be the thermodynamic minimum of the system, determined by a compromise between the entropic drive to form the smaller assembly, and the enthalpic disadvantage of pairing two bulky ligands on the same edge.

### 2.6 Multicavity cages

**Fig. 17:**
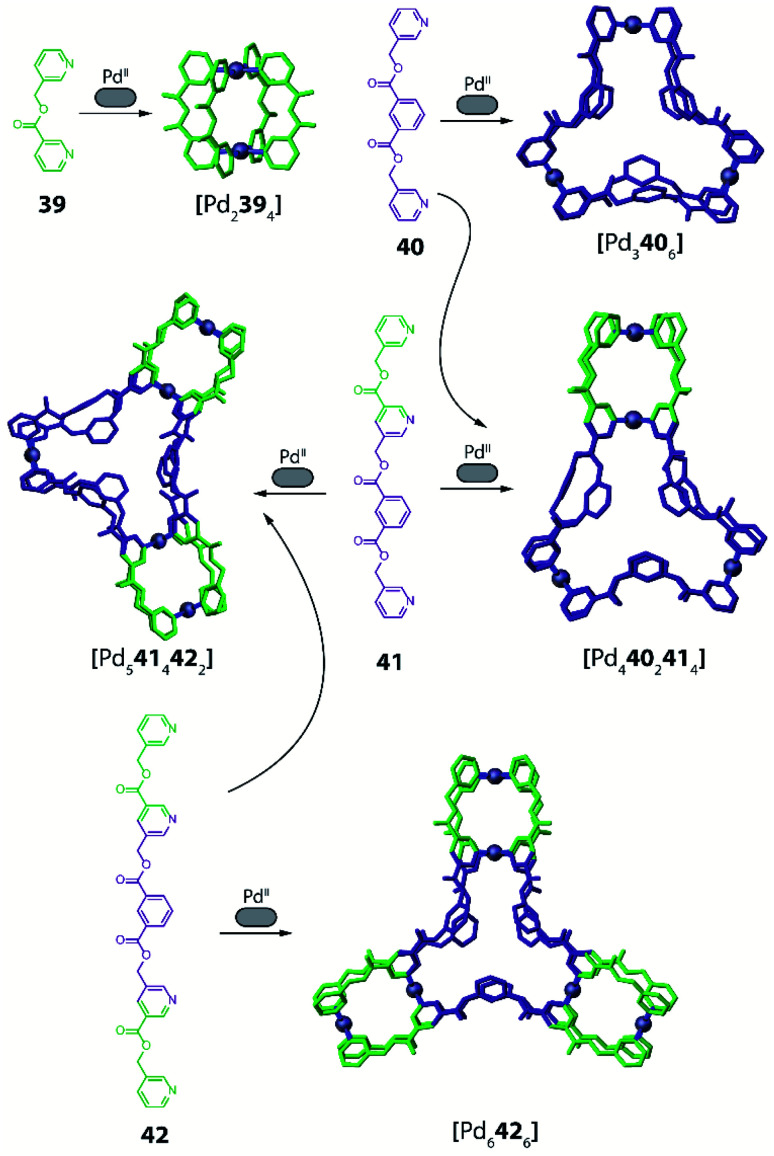
Self-assembly of Pd^II^ with bis- and trismonodentate ligands to achieve a series of homo- and heteroleptic multicavity metallo-supramolecular hosts.

A different facet of using non-symmetric ligands has been recently reported by Chand and co-workers in the course of forming Pd-mediated architectures possessing multiple cavities.^96^ The systems design relies on a series of polytopic ligands having two, three or four pyridine donor groups respectively (ligands **39–42**, Fig. 17). The multicavity systems are formally achieved from the conjunction of a central [Pd_3_L_6_] ring with peripheral [Pd_2_**L**_4_] cages through covalent extension of the ligands. Initially, the authors studied the homoleptic precursors having only one cavity. Self-assembly of Pd^II^ with **39** results in a dinuclear [Pd_2_**39**_4_] cage, while from **40** a three-membered [Pd_3_**40**_6_] ring is formed, as confirmed by crystal structures together with ESI-MS and NMR analyses (Fig. 17). Interestingly the assembly obtained with rather flexible ligand **40** is concentration dependent. While [Pd_3_**40**_6_] remains the main species, successive dilution enriches the fraction of a [Pd_2_**40**_4_] cage. On the contrary, in a more concentrated sample, even [Pd_4_**40**_8_] is detected as minor component. Self-assembly of Pd^II^ with non-symmetric, tris-monodentate **41** (formally a fusion of **39** and **40**) results in a mixture of trinuclear [Pd_3_**41**_4_] and hexanuclear [Pd_6_**41**_8_] compounds (not shown). Subsequently, ligands **40** and **41** were combined in a 2 : 4 ratio, resulting in integrative self-sorting to give a tetranuclear heteroleptic [Pd_4_**40**_2_**41**_4_] assembly possessing two different cavities able to bind small anionic guests (NO_3_^−^, F^−^, Cl^−^, Br^−^; Fig. 16).

Furthermore, a pentanuclear compound possessing three cavities was achieved by integrative self-sorting of ligands **41** and **42** with Pd^II^ cations. The resulting heteroleptic [Pd_5_**41**_4_**42**_2_] architecture resembles a conjunction of a [Pd_3_**40**_6_] ring with two [Pd_2_**39**_4_] cages, as confirmed by single crystal X-ray diffraction (Fig. 17). Finally, a three-bladed, hexanuclear structure [Pd_6_**42**_6_] with four cavities was obtained using symmetric ligand **42** alone (Fig. 17). While this compound is a homoleptic structure, as it is based on only one ligand, its structural complexity is intriguing since all vertices of a triangular core resembling [Pd_3_**40**_6_] are formally extended by a [Pd_2_**39**_4_] cage.

Formation of the two heteroleptic architectures [Pd_4_**40**_2_**41**_4_] and [Pd_5_**41**_4_**42**_2_] could also be achieved when mixing homoleptic cage [Pd_3_**41**_4_] with ring [Pd_3_**40**_6_] or [Pd_6_**42**_6_], respectively, proving the former systems to be thermodynamic products. On the other hand, when combining only the homoleptic rings [Pd_3_**40**_6_] and [Pd_6_**42**_6_], the system shows narcissistic self-sorting and no further species are observed.

**Fig. 18:**
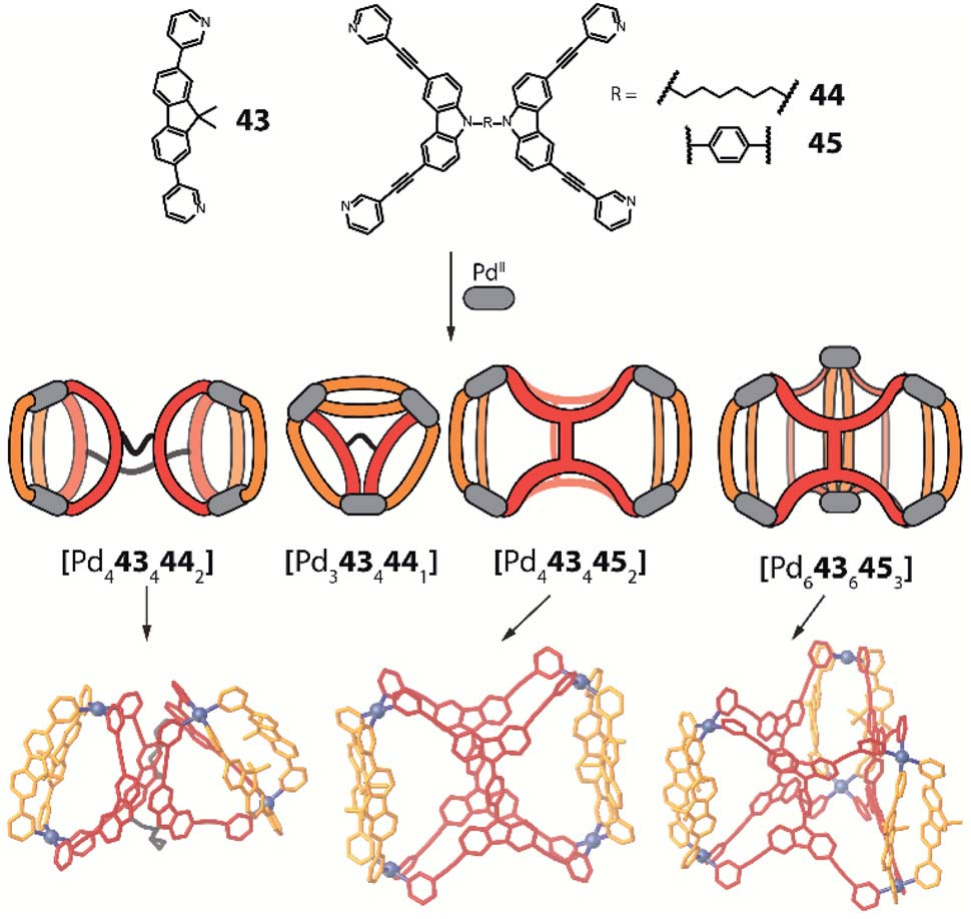
The flexibility of the linker in backbone-bridged ligands determines (together with solvent and guest effects) the formation of a variety of higher-order heteroleptic structures with very different cavity shapes and sizes.

Another approach towards multi-cavity cages was chosen by Clever *et al.* who expanded their previously reported set of shape-complementary ligands pairs^78,79^ with derivatives of dual banana-shaped bis-monodentate ligands covalently bridged *via* their backbones (Fig. 18).^97^ Based on the observation that ligand **43** cleanly forms *cis*-[Pd_2_**43**_2_**26**_2_] heteroleptic cages with above-introduced carbazole-derived ligand **26**, a series of higher-order assemblies was achieved. When **43** was mixed with dual ligand **44**, in which both carbazole sites are back-to-back connected with a flexible linker, Pd^II^ coordination in acetonitrile afforded [Pd_4_**43**_4_**44**_2_] as a formal dimer of *cis*-[Pd_2_**43**_2_**26**_2_]. Interestingly, reaction of the same components in DMSO gave trinuclear compound [Pd_3_**43**_4_**44**_1_] whose shape resembled a tetrahedron as the hexylene bridge protrudes from one face of the three-membered core. In contrast, ligand **45** featuring a rigid backbone bridge yielded a tetranuclear assembly [Pd_4_**43**_4_**45**_2_] which only resembles compound [Pd_4_**43**_4_**44**_2_] on first sight: closer inspection of its X-ray structure reveals that ligand **45** is now rotated by 90° as compared to **44**, hence the carbazole backbones are not joining the ends of the same counter ligand **43** but bridge two oppositely arranged ones (Fig. 18). Finally, the presence of sulfonate anions was found to trigger rearrangement into a [Pd_6_**43**_4_**45**_6_] barrel-shaped host upon crystallization, thus complementing the system by a structure of even higher nuclearity.

As shown in the reported examples, the use of non-symmetric ligands is a powerful tool to increment the structural complexity of coordination-driven architectures. This approach can be combined with the previously discussed coordination sphere engineering and shape complementarity approaches and can lead from heteroleptic [M_2_L_4_] cages to compounds with higher nuclearity and cavity count.

## 3. Functional homoleptic cages

Having outlined different strategies for increasing the structural complexity of self-assembled coordination cages, we will now examine how to introduce functionality in such systems. Ultimate aim is to achieve “multifunctional” systems in which several distinct chemical moieties act together in an emergent fashion to allow applications in enzyme-inspired catalysis or vectorial excitation or electron transfer. It should be noted that to date most of such functional systems are still of homoleptic nature, while heteroleptic structures exhibiting distinct functions are quite rare. Therefore, we first focus on functional homoleptic cages and continue in the final section with more sophisticated examples that are based on heteroleptic cages and rings. “Functionality” is of course a very general term and thus needs to be subclassified. In the context of this review, we distinguish between the following different tasks: (a) guest uptake and release, (b) control (or observation) by light, (c) host–guest chemistry under control of a chemical stimulus and (d) (catalytic) chemical transformation of substrates influenced by the metallo-supramolecular host.

In general, coordination cages are renowned for their rich host–guest chemistry, *i.e.* the selective uptake of small, charged species,^98–100^ or even neutral molecules.^101–105^ Formation of a host–guest complex is driven by either electrostatic interactions, such as hydrogen bonding, π-stacking, Coulomb or dispersion interactions,^102^ solvophobic effects,^106^ or a combination of these. Pd^II^-based coordination cages are usually positively charged and, thus, preferably host negatively charged species such as small anions or sulfonated organic molecules.^107^ On the other hand, Raymond's [Ga_4_L_6_]^12−^ tetrahedra^108^ with three catecholate donors at each metal centre are negatively charged, allowing for encapsulation of positively charged species. Therefore, Raymond could successfully apply this cage for several chemical reactions with positively charged intermediates,^109–111^ which are stabilized by the cage environment. Fujita designed a water-soluble cage which readily takes up neutral organic molecules due to the hydrophobic effect and allows numerous different reactions to be performed in its nano-confined cavity.^112^ Recently, Lusby reported the uptake and chemical conversion of neutral *para*-quinone into a cationic Pd^II^-cage. The main driving force here are multiple non-covalent interactions.^113^ These examples illustrate the variety of host–guest interactions and the high potential of coordination cages for applications in selective recognition and chemical transformation processes. In the past decade, Crowley, Fujita, Shionoya, Nitschke, Sallé, Clever and others have shown that host–guest chemistry can be controlled by external stimuli such as chemical triggers, redox processes, temperature, light or change of solvent. This adds additional possibilities for tuning the function and application of coordination cages. For example, Sallé and co-workers used redox agents to control the oxidation state, and hence overall charge, of their M_2_L_4_ architecture, built from ligands based on tetrathiafulvalene derivatives. They showed that control over the redox state triggers coordination cage assembly and disassembly, causing reversible binding and release of B_12_F_12_^2−^ guests.^114^

In the following, we will illustrate some examples of homoleptic cages, which integrate specific functionality with an effect on their host–guest chemistry, including control by external stimuli. Even more about this chemistry can be found in a further selection of recent reviews.^44,45,115,116^

### 3.1 Chromophore-based cages

The interaction of self-assembled structures with light can lead to a multitude of effects and applications, including cavity-confined photo catalysis,^46^ triggering of photoswitches (see below) and guest detection *via* specific spectral signatures. To the latter area, Clever and coworkers recently contributed a new family of dye-based coordination cages whose bis-monodentate ligands carry long-known coal tar dye derivatives in their backbones (Fig. 18).^117^

**Fig. 19:**
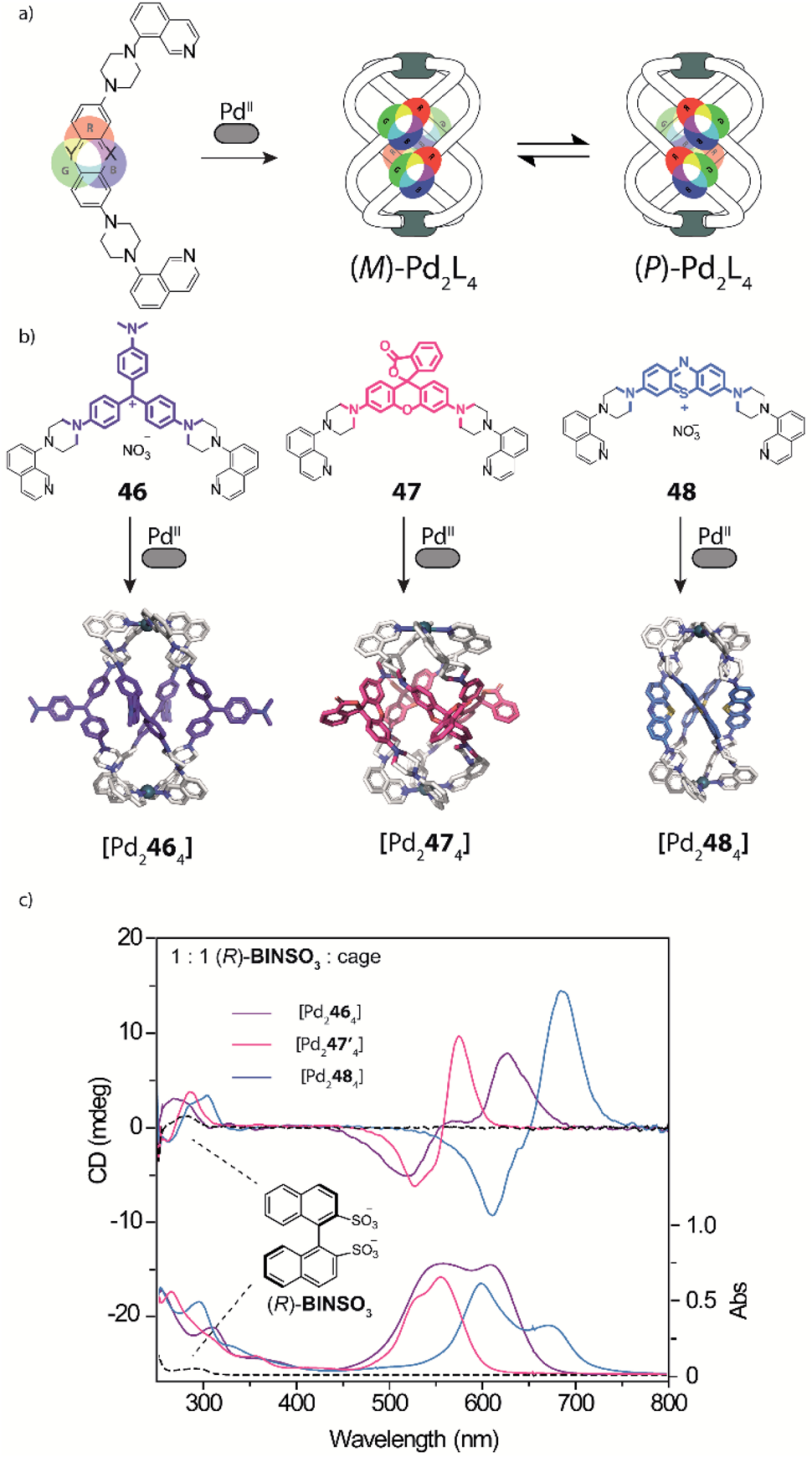
(a) and (b) Ligands with strongly coloured coal tar dyes in their backbone and quinoline donors lead to a series of chromophore-based helicates as dynamic mixture of enantiomers; (c) binding of homochiral guests shifts this equilibrium to one helical form *via* chirality transfer, leading to strong CD effects far in the visible region (**47′** = pyridine analogue of **47**).

Key structural element is further the implementation of piperazine linkers between the chromophores and coordinating donors as crucial requirement to retain the dyes' properties by serving as auxochromic groups (as they mimic the typical dimethyl amine substituents of many coal tar dyes, serving to reduce the HOMO–LUMO gap in the extended push–pull electronic system). Based on this principle, coloured helicates and cages comprising the chromophores Michler's ketone, crystal violet ([Pd_2_**46**_4_]), rhodamine ([Pd_2_**47**_4_]) and methylene blue ([Pd_2_**48**_4_]) were synthesized and fully characterized. While ligands with pyridyl donors led to the formation of typical [Pd_2_**L**_4_] cages featuring an accessible cavity (not depicted), the equipment with isoquinoline donors afforded strongly twisted helicates that rapidly convert between two enantiomeric conformers (Fig. 19a and b). This feature was then employed to examine chiral compounds (exemplified with small molecules but bearing potential for the detection of anionic bioanalytes such as DNA) through chirality transfer from the bound guest to the helicate, inducing an equilibrium shift towards one preferential enantiomeric form and hence giving rise to a strong chiroptical answer in the visible region of the circular dichroism spectrum (Fig. 19c).

### 3.2 Light as trigger in homoleptic [Pd_2_**L**_4_] cages

Light is considered as a clean “reactant”, as it can be used to precisely activate specific processes in complex mixtures, leaves no byproducts and is in principle abundantly available in the form of sunlight. Ideally, the choice of a specific wavelength allows for the directed activation of only a photoactive component without affecting or damaging other parts of a metallo-supramolecular assembly.

For example, Wezenberg and Feringa presented a photo-responsive coordination cage based on Pd^II^ cations and bidentate bis-pyridyl ligands with an overcrowded alkene as backbone.^118^ This motif is similar to their famous molecular motors^119^ and can be switched reversibly by irradiation. Irradiating the *Z*-enantiomer of the ligand (or cage) with 312 nm light at −78 °C, and then slowly warming up to room temperature, leads to a switch to the *E*-enantiomer. Further irradiation results in formation of an unstable form of the *Z*-enantiomer, which can be rotated back by irradiation with 365 nm light. All three cage photoisomers are able to bind OTs^−^ due to electrostatic interactions. Even though the cages are geometrically very different, no large difference in binding affinity of the guest was observed.

Clever has prepared ligands with photochromic dithienylethenes (DTE) as backbone and studied their self-assembly behaviour with Pd^II^ cations.^120,121^ DTE is a photo-switchable molecule that exhibits two different isomers, namely the flexible open and the rigid closed form. Under visible light, DTE predominantly adopts its open form. Irradiation with UV light (313 nm) leads to formation of the closed form *via* an antarafacial 6e^−^ electrocyclic ring closure. In 2013, a [Pd_2_**L**_4_] cage containing the DTE unit backbone and pyridine donors was shown to bind model guest compound [B_12_F_12_]^2−^ preferably in its open form while closure of all DTE switches resulted in significant reduction of guest binding affinity.^120^

**Fig. 20:**
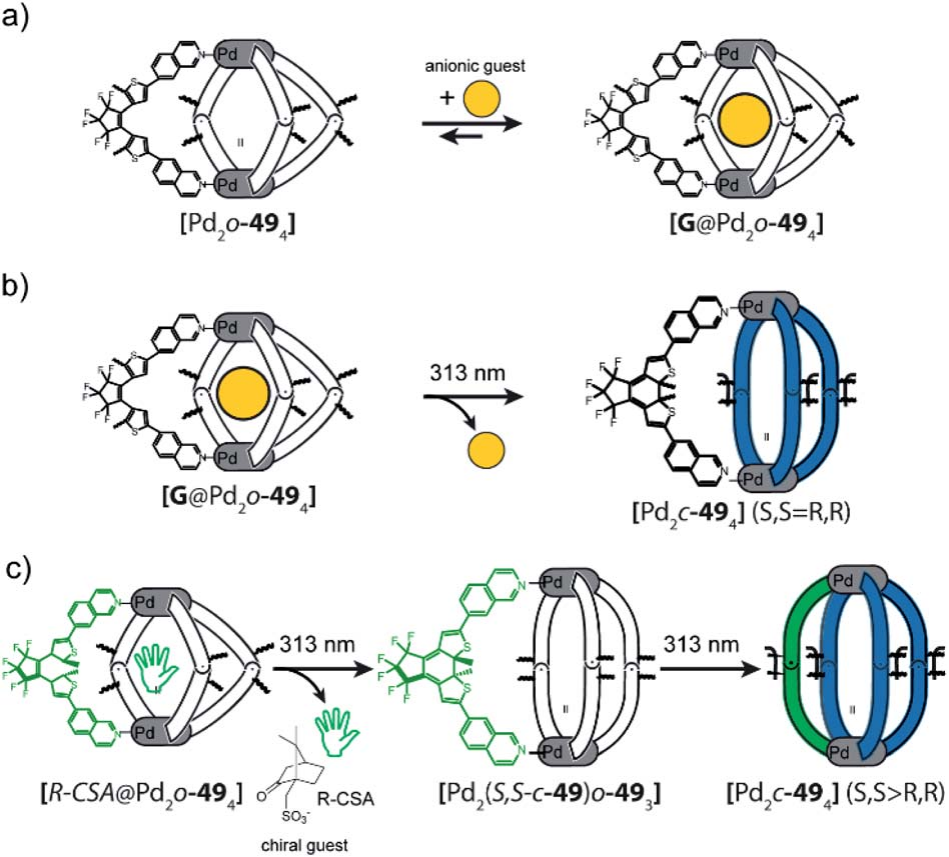
(a) Binding of anionic guests in flexible DTE cage [Pd_2_o-**49**_4_], (b) irradiation at 313 nm leads to closure of all photoswitches and generation of rigid [Pd_2_c-**49**_4_] which causes guest ejection–irradiation at 617 nm reverses this process, (c) chiral guest *R*-camphor sulfonate transfers chirality to the first ligand that is switched from the open to the closed form. After this the guest is released, the other three ligands switch without stereochemical control.

In a follow-up study, the DTE ligand was modified with two 7-*iso*-quinoline moieties as donors to form similar cages of the [Pd_2_**L**_4_] type, both in the open (o-**49**) and closed (c-**49**) form (Fig. 20).^122,123^ [Pd_2_o-**49**_4_] can bind a chiral guest molecule, such as *R*- or *S*-camphor sulfonate (CSA). While the cage itself, composed of *C*_2_-symmetric but quickly epimerizing DTE backbones, does not show any chiroptical effects, the host guest-complex with enantiopure CSA shows a strong guest-induced circular dichroism (CD) effect in the absorption regime of DTE chromophore. This is a clear indication for chirality transfer from the guest to the host. This finding was supported by a competition guest binding experiment: when 1,4-benzenesulfonic acid, a stronger binding guest, is added to the mixture, CSA is released, and the CD effect is cancelled. Guest ejection is also observed upon irradiation (313 nm), which results eventually in formation of [Pd_2_c-**49**_4_] with all four photoswitches closed. Similar to the previously reported example, the anionic guest does not bind to [Pd_2_c-**49**_4_]. The question arose at which step in the successive photoclosure of the four switches the guest is ejected. This could then be answered by a combination of CD and ion mobility mass spectrometry experiments, supported by following the fate of chiral information transfer throughout the irradiation process by analyzing the enantiomeric excess of (configurationally stable!) closed DTE ligand after cage disassembly.^122^ Apparently, the guest is already ejected after switching of the first ligand, pointing to a situation in which the rigid, closed ligand photosiomer dictates the conformational preference of the remaining open ligands. This assumption was further supported by comparing X-ray structures of cage isomers with four open, two open/two closed and four closed switches^123^ and recently supported through MD simulations and detailed binding enthalpy/entropy calculations by Schäfer *et al.*^124^

### 3.3 Small anions as chemical trigger

**Fig. 21:**
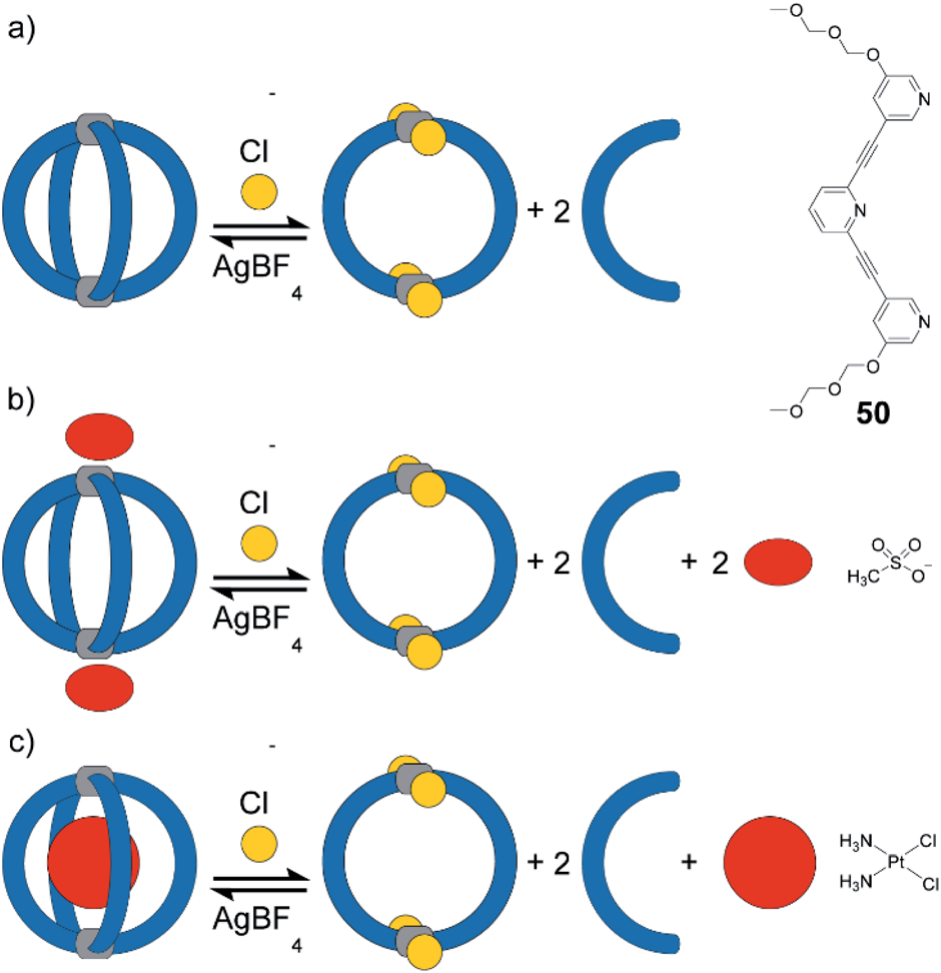
(a) Reversible switch between [Pd_2_**50**_4_] and [Pd_2_**50**_2_Cl_4_] in acetonitrile or DMF upon addition/sequestration of chloride; (b) mesylate guest binds exohedrally to [Pd_2_**50**_4_] and is released upon addition of chloride; (c) cis-platin binds in the cavity of [Pd_2_**50**_4_]. Formation of the ring releases the guest.

Instead of light as external stimulus, also chemical compounds can be added to a supramolecular cage to affect its host–guest chemistry. Crowley exploited the dynamic nature of Pd^II^-coordination chemistry and demonstrated the reversible transformation of a [Pd_2_**50**_4_] cage to a [Pd_2_**50**_2_Cl_4_] ring upon addition of chloride anions under substitution of two bridging ligands per cage (Fig. 21).^125^ Diglyme substituents at **50** ensured sufficient solubility of the released ligands. Consequently, after addition of AgBF_4_ and precipitation of AgCl, ligands are picked up again and [Pd_2_**50**_4_] cage is readily reformed. Interestingly, ring [Pd_2_**50**_2_Cl_4_] cannot be formed directly from free **50** and [Pd(CH_3_CN)_2_Cl_2_]. Instead, polymeric structures are formed. This finding points to the fact that pre-organization in form of the cage and subsequent release of two ligands is an elegant strategy to obtain such kind of ring structures (compare triple catenated rings [Pd_2_**26**_2_Cl_4_]_3_ formed from somewhat larger ligand **26** under similar conditions^80^). After establishing reversible chloride-triggered structural change from cage to ring, Crowley and co-workers tested the host–guest behaviour of the cage. They found that anionic guest mesylate binds in an exohedral fashion *via* electrostatic interaction with the Pd^II^ centres (Fig. 21b). In acetonitrile as solvent, the neutral cis-platin drug compound binds to the interior cavity, partly due to dispersion interactions. When changing the solvent to DMF, binding becomes significantly weaker, because the solvent molecules act as competing guests. Both mesylate and cis-platin are released when the cage is transformed to its ring successor. This study is a nice example of chemically driven guest-release and shows potential application in drug-delivery.

**Fig. 22:**
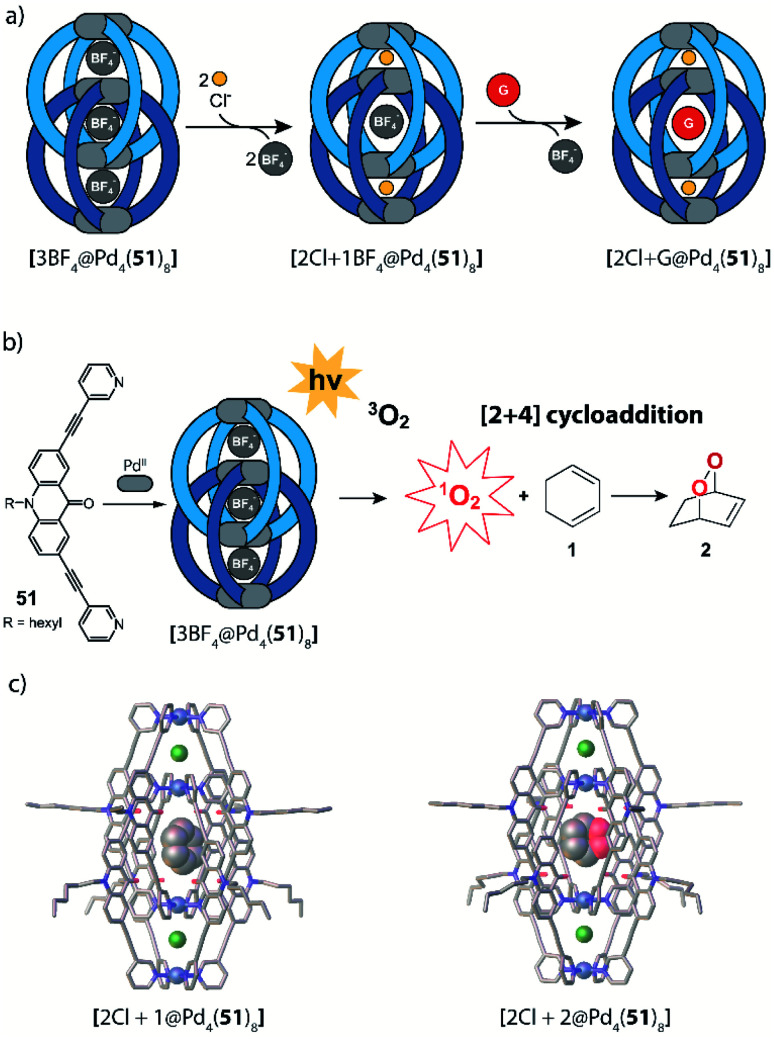
(a) Chloride-triggered uptake of neutral guests by a coordination cage [Pd_4_**51**_8_] following an allosteric mechanism; (b) both [3BF_4_@Pd_4_**51**_8_] and [2Cl + **1**@Pd_4_**51**_8_] act as photosensitizers for singlet oxygen generation. Activated oxygen adds in a [2 + 4] cycloaddition to the substrate in the cavity and surrounding solution; (c) crystal structures of [2Cl + **1**@Pd_4_**51**_8_] and [2Cl+**2**@Pd_4_**51**_8_].

In 2015, Clever and co-workers demonstrated allosteric binding of neutral guest molecules inside catenated double cage [Pd_4_**51**_8_] with chloride anions serving as chemical trigger (Fig. 22a).^101^ Starting from interpenetrated dimer [3BF_4_@Pd_4_**51**_8_] encapsulating three BF_4_^−^ counter ions, addition of chloride leads to exchange of the two BF_4_^−^ guests in the outer pockets and simultaneous enlargement of the inner pocket. This triggers neutral guests such as benzene, cyclohexane, norbornene or other molecules of similar dimensions to be bound to form host–guest complexes of the formula [2Cl + X@Pd_4_**51**_8_] (X = neutral guest). Systematic determination of binding thermodynamics by NMR titrations as well as electronic structure calculations revealed that London dispersion effects play an important role for guest uptake.^102^ Very recently, Clever and co-workers found that [3BF_4_@Pd_4_**51**_8_] and [2Cl@Pd_4_**51**_8_] act as photosensitizers for singlet oxygen generation (Fig. 22b). While the acridone-based ligand **51** alone is unstable and decomposes under irradiation in air, its assembly to supramolecular cages stabilizes the aromatic core and thus allows for its robust photosensitizing function. In the reported system, 1,3-cyclohexadiene was used in a model reaction to undergo [2 + 4] cycloaddition with singlet oxygen generated by energy transfer from the excited cage species to ^3^O_2_. While it is worth noting that the substrate could be encapsulated inside the cavity of photocatalytic [2Cl@Pd_4_**51**_8_] singlet oxygen sensitization and hetero-Diels–Alder reaction can also take place on the outside, as even [3BF_4_@Pd_4_**51**_8_] shows activity despite its inability to act as host for the substrate. Encapsulation of both substrate and endoperoxide product in [2Cl@Pd_4_**51**_8_] was confirmed by NMR, MS and X-ray techniques (Fig. 22c).^126^

### 3.4 Temperature as external stimulus

**Fig. 23:**
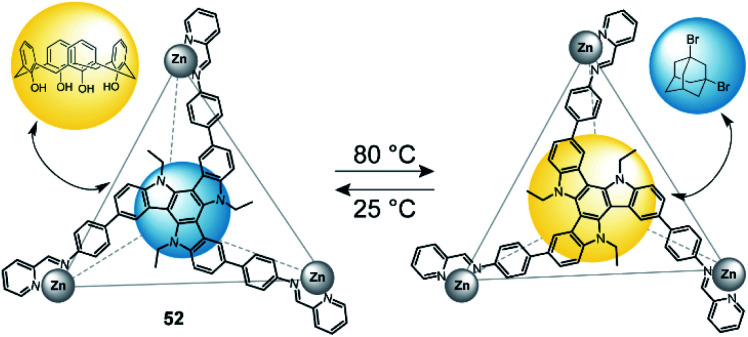
Temperature-controlled selectivity of guest-uptake in a triazatruxene-faced zinc tetrahedron.

In 2019, Nitschke and co-workers reported a triazatruxene-faced (**52**) tetrahedral coordination cage assembled through Zn^II^ cations.^127^ This cage can adopt two diastereomeric configurations (*T*_1_ and *T*_2_), which can be interconverted by a change in temperature (Fig. 23). *T*_1_ is favoured at lower temperatures, while *T*_2_ is predominant at temperatures above 80 °C. The authors utilized this circumstance and tested guest-binding affinity of a variety of compounds. It turned out that *T*_1_ preferably binds small aliphatic guests such as dibromoadamantane. *T*_2_ on the other hand readily binds larger aromatic guests such as calix[4]arene. The different preference is a result of different cavity sizes of *T*_1_ and *T*_2_.

### 3.5 Catalysis inside homoleptic coordination cages

The abovementioned examples illustrate the rich host–guest chemistry of many homoleptic coordination cages. By installation of responsive units such as photoswitches into the ligands, host–guest chemistry can be controlled by external stimuli. This can be simply the release (or uptake) of one specific guest. More complex systems can distinguish between a mixture of substrates and pick out the best-fitting guest. The nature of the cage plays an important role in guest binding. So are cationic cages quite likely to bind negatively charged guests. And hydrophobic cavities will bind unpolar guest much stronger in aqueous media than in organic solvents.

Selective guest binding is often a precondition for subsequent chemical transformations inside the cavity. In terms of catalytic reactions, supramolecular chemists like to compare their artificial systems to natural enzymes.^33,34,47^ Active sites in enzymes are often buried within a protein, creating a hydrophobic cavity. Early reports on artificial enzyme mimics utilized highly polar, water-soluble compounds such as cyclodextrins^128^ or compounds with large aromatic panels for substrate binding.^129^ A typical reaction that was explored with these systems is the Diels–Alder reaction.^130^ Catalytic activity for bimolecular reactions is often driven by an increase of the local concentration of reaction partners within the microenvironment created by the cavity. In the following, we will discuss some more recent literature on cage catalysis, where external stimuli play an important role. The scope of this review does not allow to cover all literature on cage catalysis. We rather pick out a few outstanding examples and ask the interested reader to refer to recent reviews in this area.^34,46,47,131,132^

**Fig. 24:**
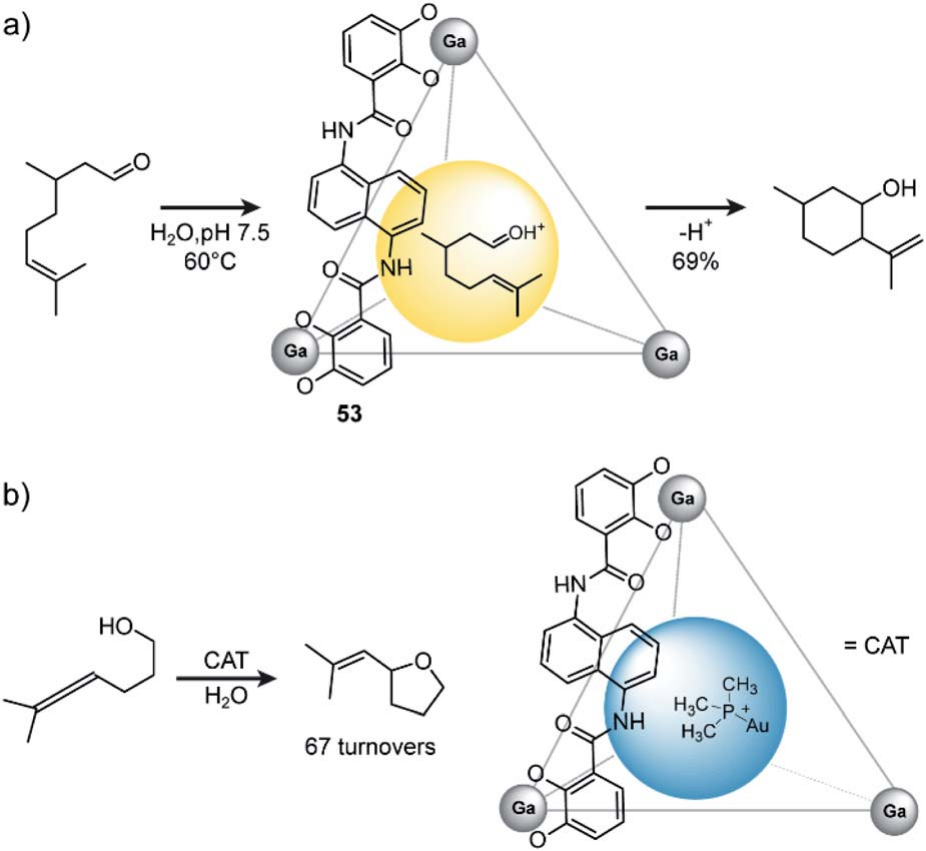
(a) Prins cyclisation of citronellal catalysed by Raymond's Ga^III^ coordination cage. (b) Encapsulated catalyst Me_3_PAu^+^ catalyses hydroxyalkylation of alcohol-tethered allenes. Reproduced from ref. 137 with permission from the Royal Society of Chemistry, copyright 2021.

In general, there are three different scenarios for cage catalysis: (i) the supramolecular host encapsulates substrates and thus increases local concentration, (ii) a catalyst is co-encapsulated together with substrates, (iii) components of the cage (either ligand or metal) possess a distinct function and participate actively as catalysts in the reaction. Toste, Raymond and Bergman utilized their aforementioned homoleptic Ga^III^-based cage^108^ with bis-bidentate catechol ligands **53** to accelerate a variety of reactions (Fig. 24). In early studies, they found that the cage promotes formation of iminium cations^133^ and protonation of amines in aqueous media.^134^ On the one hand, the hydrophobic cavity encapsulates these products, shields them from bulk water and protects the cationic species. On the other hand, electrostatic interactions between the anionic cage and cationic guest stabilize the latter. The authors went on and studied the performance of the cage in catalytic reactions. As an early example, they demonstrated successful catalysis of the Prins cyclization of citronellal (Fig. 24a).^135^ In comparison to the same reaction in bulk solution, where several side products were observed, with the cage serving as catalyst the expected product formed in 69% yield as major species.

Concerning the encapsulation of a catalyst in the cavity, Raymond and coworkers bound gold complex Me_3_PAu^+^ inside their cage. This host–guest complex catalysed the hydroxyalkylation of alcohol-tethered allenes with up to 67 turnovers (Fig. 24b).^136^

In a similar fashion, Reek utilized Fe^II^-based cages built from Zn^II^-porphyrines for encapsulation of a variety of catalysts.^138–140^ In these cages, the Zn^II^-porphyrine-based ligands play a dual function: with their four terminal diimine donors, they coordinate the Fe^II^ vertices, and the central Zn^II^-cation contributes to catalyst encapsulation by coordinating the pyridine-donors included in the ligands of the catalyst. Especially for reactions in which the redox state and thus, the charge and coordination ability of the catalyst are changing, such additional anchors are quite important. In a recent study, Reek encapsulated a [FeFe] hydrogenase active site model compound with the aid of a pyridyl-phosphole ligand as anchor into such a cage.^139^ This combination drives the photochemical proton reduction. In an electrochemical follow-up study,^138^ the same authors utilized a slightly modified version of the diiron complex and identified a shift to lower overpotential of the [FeFe] catalyst as a result of encapsulation inside the cationic cage.

The third scenario for cage-catalysis is the utilization of reactive or light-susceptible components for cage assembly. Strictly speaking, already Reek's porphyrin cage belongs to this category, because the porphyrin ligands can act as photosensitizers. In another example, Su and coworkers prepared a Pd^II^-connected octahedron with Ru-tris-phenanthroline-based tris-monodentate ligands.^141^ Under irradiation and in presence of triethylamine, the authors observed hydrogen evolution.^142^ In this system, the metallo-ligands function as photosensitizers, while the Pd^II^-nodes have a dual role: they act both as mediators for supramolecular assembly as well as active sites for proton reduction.

## 4. (Multi)functional heteroleptic cages

While Section 2 illustrates that the structural complexity of supramolecular architectures has already evolved significantly in terms of heteroleptic assembly, the controlled introduction of functionality into these systems is still in its infancy. Possible applications of such multifunctional coordination cages include modular receptor platforms, efficient excitation- or charge-transfer systems based on donor–acceptor combinations or cooperative catalysts. Similar to many enzymes, such systems could include a receptor that is reacting to an external input such as light, change in pH or the addition of a chemical substance. Activation of the supramolecular system by an external stimulus could then cause a response such as structural rearrangement, change in guest-binding ability, charge-transfer between two components or the promotion of a chemical reaction. In the following, we will discuss recent studies from the literature, where two or more different ligands, comprising at least one functionality, have been combined in the form of one heteroleptic coordination cage.

### 4.1 Guest-inclusion by heteroleptic and pseudo-heteroleptic cages

**Fig. 25:**
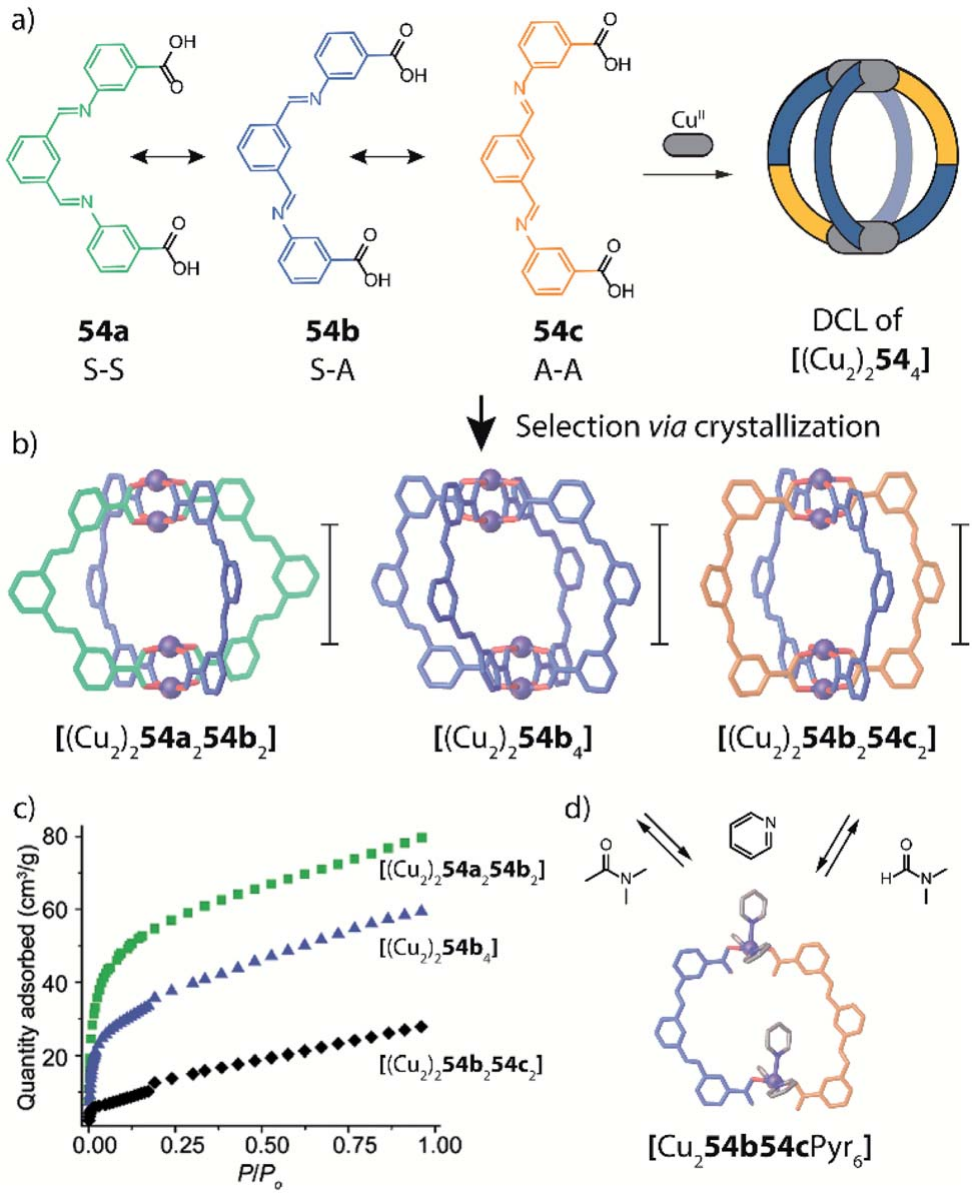
(a) Ligand conformers **54a–c** assemble with Cu^II^ cations to a library of cage isomers [(Cu)_2_**54**_4_]; (b) crystallization conditions allow selection of specific conformers which (c) show different CO_2_ uptake characteristics and can be reversibly converted into (d) dinuclear rings by pyridine addition.

In the first example, reported by Bloch and co-workers, a diamine ligand **54** was used that can interconvert between three different backbone conformers, giving rise to formation of a dynamic combinatorial library (DCL) of Cu_2_-based cage isomers [(Cu)_2_**54**_4_] of different shape (Fig. 25a).^143^ The authors found that differing the solvent compositions used for crystallizing the metallo-supramolecular assemblies allowed to select distinct solid state morphologies, differing in their composition of ligand isomers and including pseudo-heteroleptic species, where two different ligand isomers are present in the same species (Fig. 25b). Furthermore, CO_2_ uptake was studied in activated powders of the different isomeric materials, showing that gas uptake characteristics are significantly modulated, despite the fact that the only differences between the constituents are the ligands' conformational features (Fig. 25c). Finally, it was shown that the cages [(Cu)_2_**54**_4_] can be transformed into dinuclear rings when ligands are partially replaced by added pyridines (Fig. 25d).

In 2015, Yoshizhawa showed a nice example of guest-induced heteroleptic cage formation.^144^ If two homoleptic coordination cages based on anthracene-containing ligands with phenylene- and naphthalene backbones, respectively, are mixed in DMSO, dynamic ligand exchange results in a statistical mixture of cages 
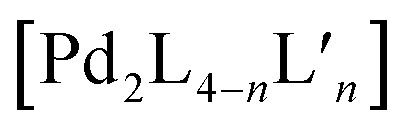
 (*n* = 0–4). Addition of fullerene C_60_ allows to drive the dynamic equilibrium efficiently to form only the host–guest complex 
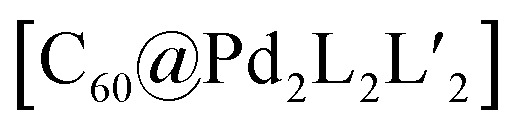
 as single product. The phenylene-based homoleptic cage alone showed to be too small to encapsulate C_60_, while the larger naphthalene-based cage alone prefers to bind larger guests such as C_70_ or malonate-C_60_. Guest binding in this system is driven by π,π-interactions between the large aromatic panels on the ligands and the fullerene guest. It seems that the heteroleptic C_60_ complex shows the best host–guest match of π-faces, and thus is the most stable form.

**Fig. 26:**
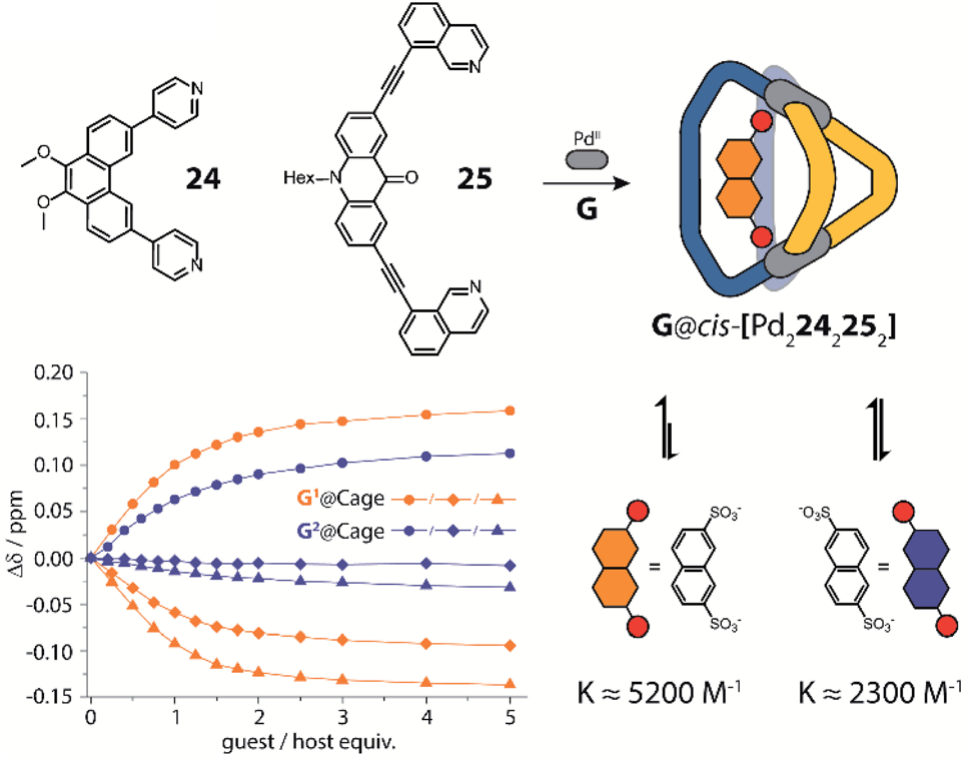
A heteroleptic coordination cage distinguishes between two different guests based on shape-selectivity.

As discussed above, heteroleptic coordination cages can also be formed selectively without the use of a template. Clever's heteroleptic cage *cis*-[Pd_2_**24**_2_**25**_2_] was prepared based on shape complementarity.^78^ The bent architecture of this host, with Pd(pyridine)_4_ planes tilted with respect to each other, encouraged the authors to test whether it could be utilized for shape-specific guest binding. Indeed, ^1^H-NMR titration revealed that a bent 2,7-naphthalene disulfonate guest binds more than twice as strong as its linear 2,6-analogue (Fig. 26).

The clear preference for the bent guest demonstrates nicely how a low-symmetric coordination cage can selectively choose a guest of matching shape. Interestingly, opposite guest selectivity was observed when letting the same guests compete for binding inside a *D*_4h_-symmetric [Pd_2_**L**_4_] cage with coplanar Pd(pyridine)_4_ planes, caused by a better match of the linear guest to the cylindrical cavity of a more regularly-shaped host.^145^

**Fig. 27:**
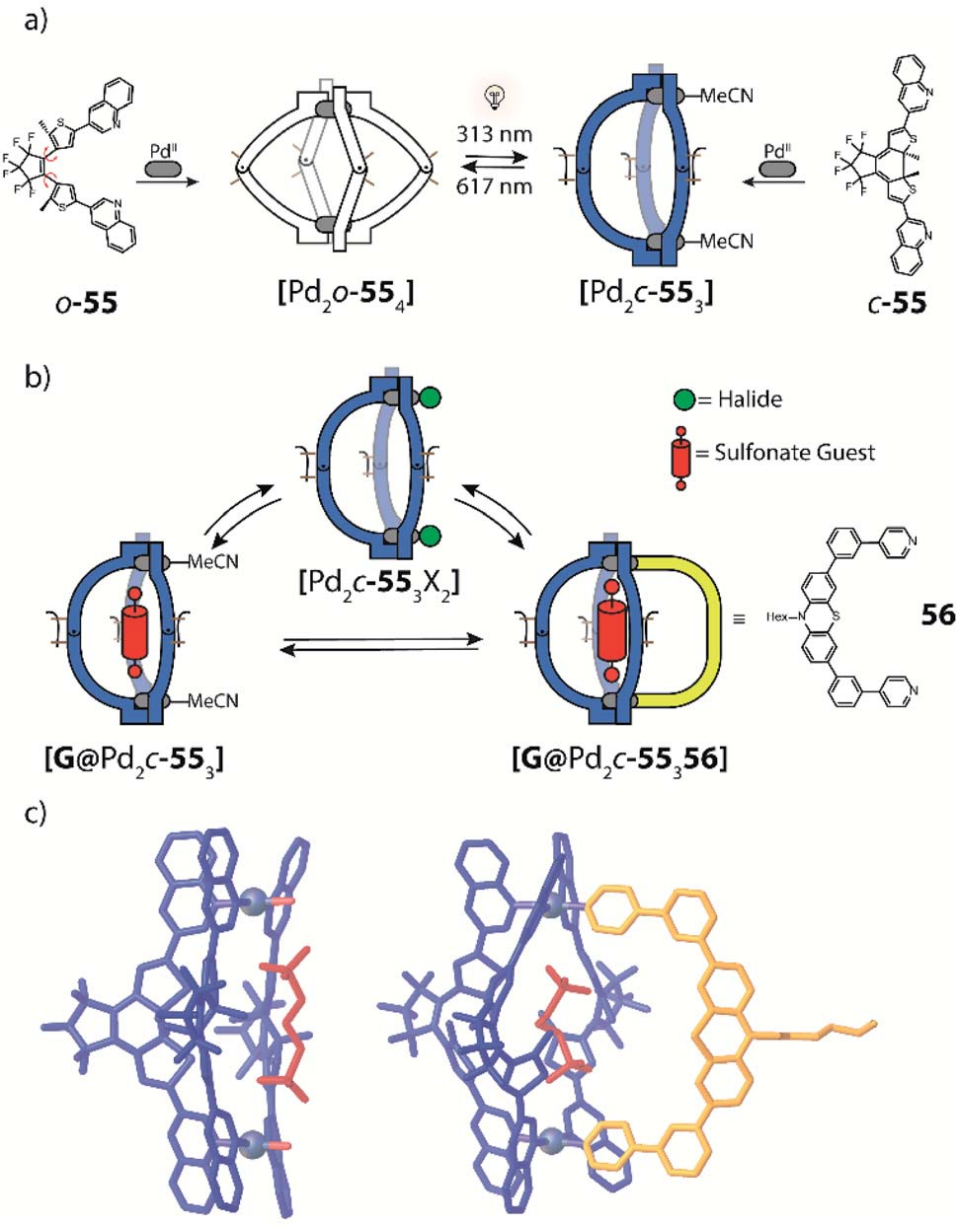
(a) Photoswitching a quinolin-substituted ligand with dithienylethene (DTE) backbone allows cycling between a [Pd_2_**L**_4_] cage and [Pd_2_L_3_Solv_2_] bowl-shaped structure; (b) guest binding can be modulated by changing the host's charge from 4+ to 2+ by adding halide anions as trigger or by changing the cavity size *via* a fourth ligand (yellow); (c) X-ray crystal structures of host–guest complexes of the bowl and unprecedented [Pd_2_L_3_L] heteroleptic cage.

A significantly more intricate system that accepts the action of three different inputs to control the guest binding ability of a series of chemically related hosts was recently reported by the same authors. Here, photoswitchable ligands based on DTE backbones, as introduced above, were employed, but this time equipped with sterically demanding quinoline donors that were discussed in the ‘coordination sphere engineering’ approach described in Section 2.2 (Fig. 27a).^146^ Photoisomerization in this system is now able to tip the balance between formation of a [Pd_2_o-**55**_4_] cage with four open photoswitches and a [Pd_2_c-**55**_3_Solv_2_] bowl containing only three DTE bridges (as closed form) for steric reasons and two solvent molecules that complement the square-planar Pd^II^ coordination spheres. While guest uptake behaviour already differs between the cage and the bowl, the latter one could be modulated in its host properties by applying two further stimuli (Fig. 27b). On the one hand, replacement of the coordinated solvents by halide anions changes overall charge from 4+ to 2+, thus decreasing the affinity for uptake of an anionic guest. On the other hand, a fourth ligand carrying less bulky pyridine donors (such as **56**) could be installed to obtain an unprecedented heteroleptic [Pd_2_c-**55**_3_**56**] motif containing two different ligands in a 3 : 1 ratio within one dinuclear cage. Again, this cage's host properties differ from the parental bowl and could be further tuned by substituting **56** with other bridges of different steric demand. Host–guest complexes of bowl [Pd_2_c-**55**_3_Solv_2_] and heteroleptic cage [Pd_2_o-**55**_3_**56**] could be characterized by single-crystal X-ray structures (Fig. 27c).

### 4.2 Light-induced charge transfer in heteroleptic donor–acceptor systems

**Fig. 28:**
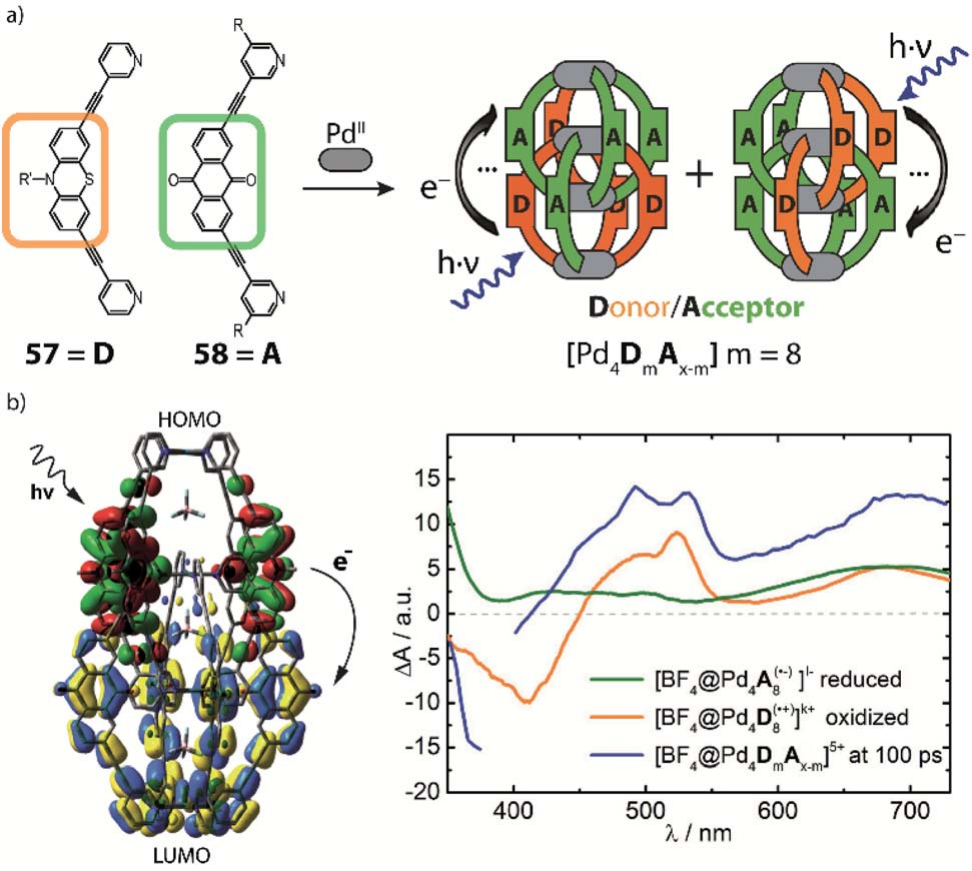
(a) Phenothiazine-based donors (D) and anthraquinone-based acceptors (A) were implemented in ligands whose combination with Pd^II^ cations leads to a statistical mixture of mixed-ligand interpenetrated double-cages which show (b) light-triggered charge separation as monitored by transient absorption and spectro-electrochemistry.

Clever and co-workers reported on the statistical assembly of mixed donor–acceptor-functionalized interpenetrated double cages [Pd_4_D_*n*_A_8−*n*_] (**57** = D, **58** = A) with electron-rich phenothiazine (D) and electron-deficient anthraquinone (A) as backbones of the ligands.^147,148^ Pd^II^-mediated assembly of ligand mixtures led to the formation of statistically mixed interpenetrated double cages, in which a varying number of D and A moieties (eight in total) are densely packed (Fig. 28). In addition, homoleptic cages of each functional ligand were prepared. Mixing both homoleptic double cages with each other did not result in ligand shuffling within several weeks, thus the system seems to be kinetically inert. Both donor- and acceptor cages show reversible electrochemical redox events at comparable potentials as the free ligands. The fate of photoexcitation within the statistically mixed heteroleptic cages was analysed by femtosecond time-resolved UV-vis and IR spectroscopy. The formation of charge separated states with a lifetime between few ps to >1.5 ns was observed. The broad range is a consequence of a statistical distribution of D and A. In contrast, mixtures of the two homoleptic cages [Pd_4_D_8_] and [Pd_4_A_8_] did not yield such charge-separated states. The drawback of working with statistical mixtures of mixed-ligand cages is a prime motivation to realize comparable donor–acceptor systems, but under full control of stoichiometry and stereochemistry. Here, strategies introduced in Section 2 promise to deliver attractive solutions.

Recently, Hai-Bo Yang and co-workers realized an interesting ring system containing a diarylethene acceptor moiety in combination with an iridium phenyl–pyridine complex as donor.^83^ Both functions are connected by *trans*-protected Pt^II^ units to form a rhombic metallacycle. The iridium complex is known to emit light of 630 nm after being excited at 380 nm. In its closed form, diarylethene serves as Förster resonance energy transfer (FRET) acceptor and thus quenches the light-pumped emission of the Ir complex. In its open form, the system shows a differing photophysical behaviour.

### 4.3 Heteroleptic cages for catalysis

**Fig. 29:**
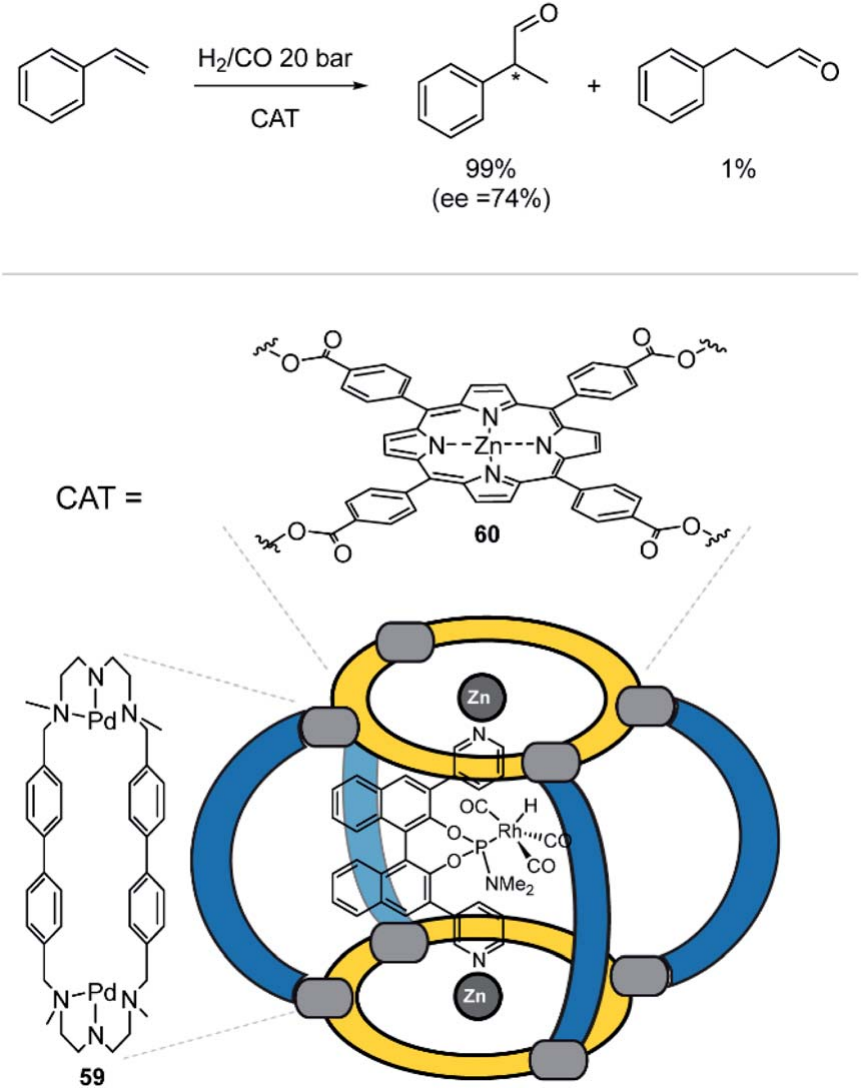
A heteroleptic coordination cage that contains a hydroformylation catalyst. The assembly drives transformation of styrene selectively to the branched product with an ee of 74%. Reproduced from ref. 137 with permission from the Royal Society of Chemistry, copyright 2021.

In 2015, Ribas, Reek and co-workers prepared a heteroleptic supramolecular box based on the charge-separation approach. The box is consisting of two Zn^II^-porphyrin-ligands **60** with four terminal carboxylate groups and four biphenyl-based amine macrocycles **59** coordinating two Pd^II^ cations, each (Fig. 29).^149^ In these boxes, the two porphyrin ligands are on opposite sites, allowing the encapsulation of a Rh-catalyst (thus giving not only a heteroleptic, but even hetero-trimetallic system under orthogonal control of all coordinative interactions!). Similar to the previously discussed homoleptic systems, the catalyst contains two terminal pyridine donors which coordinate to the Zn^II^ cations. Encapsulation of the catalyst allows for enantioselective hydroformylation, with better performance than the comparable homogenous catalyst.

Reek and co-workers also introduced Au-catalysts as endohedral substituents in M_12_L_24_ nanospheres, either covalently bound^150^ or through non-covalent interactions^151^ reaching an extreme local concentration of catalyst inside the sphere cavity. Another example of heteroleptic assemblies for homogeneous catalysis was reported by Mukherjee in 2016, where a molecular prism assembled from urea-based ligands allowed to perform catalytic Diels–Alder and Michael reactions.^152^

### 4.4 Implementing heteroleptic cages in materials

**Fig. 30:**
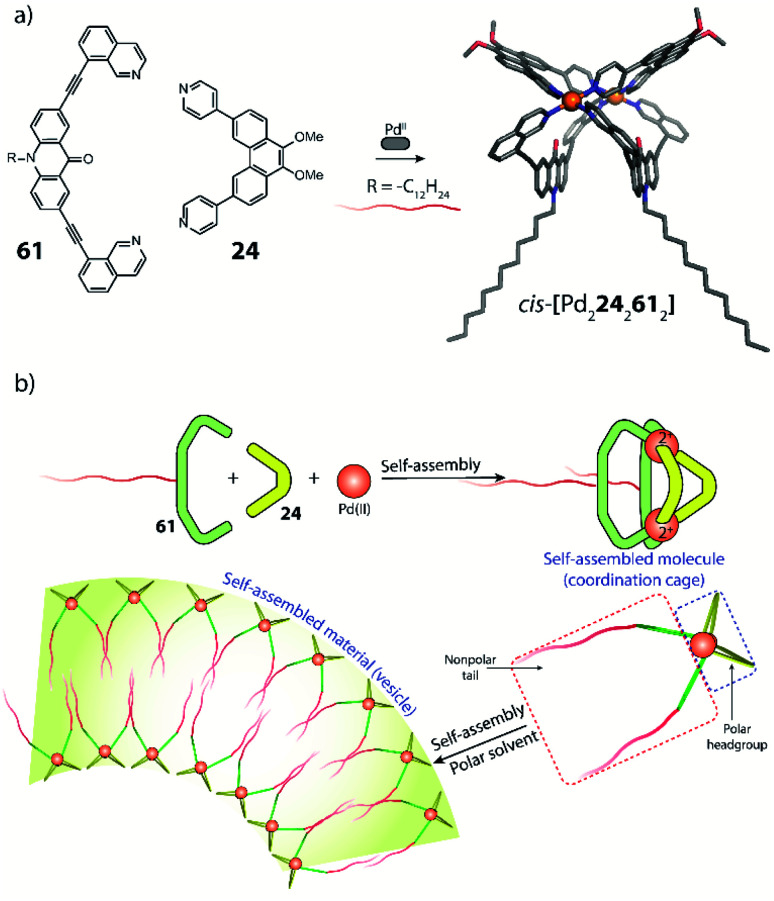
Heteroleptic cages carrying long alkyl chains act as amphiphiles to form vesicles and organic emulsions. Reproduced from ref. 153 with permission from the American Chemical Society, copyright 2018.

Finally, heteroleptic supramolecular systems can also be used as building blocks for designing novel materials. In a recent study by the Clever group, a modified version of the previously described heteroleptic cage *cis*-[Pd**24**_2_**61**_2_] with acridone (**61**) and phenanthrene (**24**) backbones was employed.^153^ The acridone ligand was equipped with a dodecyl-chain in order to increase its hydrophobicity. The phenanthrene ligand carries rather polar methoxy substituents close to the charged cage core. The heteroleptic cage that is assembled *via* shape complementarity therefore possesses amphiphilic character (Fig. 30). As shown by transmission and cryo electron microscopy, supramolecular vesicles are formed in polar organic solvents. These vesicles were shown to stabilize oil-in-oil emulsions (*e.g.* hexadecane-in-acetonitrile) for considerable times, that are usually based on solid-particle Pickering reagents. As two different organic phases can be compartmentalized on a microscopic level, these new amphiphiles promise application for the emulsion-based synthesis of structured inorganic or polymeric materials.

In a similar fashion, Stang and coworkers prepared a heteroleptic double metallacycle by using integrative self-sorting as strategy.^154^ They synthesized two ligands of different size, one with a hydrophobic side chain, the other one with a hydrophilic side chain. In addition, they prepared two pieces matching in size to each of the two dendrons and joined them with a bridge. By assembling these three building blocks *via* metal coordination, a two-faced Janus-type bicycle with amphiphilic character was obtained. In aqueous media, these supramolecules aggregated to form fiber-like structures. In contrast, spheric nanoparticles were formed in organic solvents.

## 5. Conclusions and outlook

The field of metallo-supramolecular assembly has shown that a large variety of complex nano structures can be synthesized from easily available organic building blocks and metal cations from the main or transition group elements. Most examples reported to date are based on several counts of one type of ligand, joined by one type of metal cation per assembly. Early work centred around structural characterization and simple host–guest processes. The aims of recent developments are the desymmetrization of assemblies by the non-statistical incorporation of multiple different building blocks and the introduction of function such as stimuli response, photo-redox behaviour or catalytic activity. One of the largest challenges is to combine features of both of these advances, namely the introduction of multiple functions *via* the rational integration of more than one organic bridge. Interest in such multifunctional, heteroleptic self-assembled systems is increasing dramatically. There is already a handful of strategies that allow for the non-statistical assembly of well-defined non-symmetric structures comprising multiple components. However, the major challenge is still to breathe life into these systems and make them as powerful as the complex nano systems found in nature, such as enzymes or light-harvesting structures. Furthermore, current developments will deliver tools to develop advanced functional materials.

Coordination-driven heteroleptic architectures with confined cavities are of particular interest, as they enable selective encapsulation of guest molecules, can help to activate them or stabilize labile intermediates. In this way, reactivity can be altered with respect to classic reaction media and solvent effects can be controlled within the nanoconfined environments.

In this review, we describe powerful strategies allowing to increase the structural and functional complexity of coordination-driven cages, both homoleptic and heteroleptic ones. We explored topologies where the same ligand occupies different positions in a single assembly and introduced complementary strategies for assembling heteroleptic cages, often in nearly quantitative yields. Examples based on “coordination sphere engineering” (CSE), “shape complementary assembly” (SCA), the use of non-symmetric ligands and backbone-centred steric hindrance are discussed. We want to emphasize that these approaches do not exclude each other and first examples already showcase the combination of two or more of these strategies in a single assembly.

At the same time, selected examples comprising the successful introduction of diverse types of functions have been reported. Ranging from photoswitches, strongly coloured chromophores over guest-triggered elements to specific catalysts, their implementation into self-assembled cages opens potential for stimuli-responsive guest recognition, enabling uncommon reaction pathways and harvesting solar energy. Up to date, only few examples exist where two or even more functions have been combined in a single assembly. With this work we aimed at providing an overview of the most relevant tools for non-statistical assembly and implementation of functionality. We envision that future research in this direction will lead to a better understanding of these and further assembly strategies and allow the rational generation of emergent behaviour from the interplay of different functions within nano-sized multi-component systems. This will lead to the development of novel tools for applications in research areas such as nano-medicine, sustainable synthesis, molecular electronics and intelligent materials. Inspired by the structural and functional complexity of biological structures, synthetic chemistry is stepwise increasing its control over shaping matter on the nanoscale. Bottom-up supramolecular assembly has the power to contribute strategies and innovation to translate fundamental research into value creating applications in the coming decades.

## Author contributions

All authors contributed to composing the review and preparing the figures.

## Conflicts of interest

There are no conflicts to declare.
